# Biodegradable Plastics as Sustainable Alternatives: Advances, Basics, Challenges, and Directions for the Future

**DOI:** 10.3390/ma18184247

**Published:** 2025-09-10

**Authors:** Eunbin Hwang, Yung-Hun Yang, Jiho Choi, See-Hyoung Park, Kyungmoon Park, Jongbok Lee

**Affiliations:** 1 Department of Biological and Chemical Engineering, Hongik University, Sejong 30016, Republic of Korea; c4393201@g.hongik.ac.kr (E.H.); jihochoi@hongik.ac.kr (J.C.); 2 Department of Biological Engineering, Konkuk University, Seoul 05029, Republic of Korea

**Keywords:** biodegradable plastics, biomass-derived polymers, bioplastic engineering, circular economy, sustainable materials, bio-based monomers

## Abstract

This review explores the current state and future potential of bioplastics as sustainable alternatives to conventional fossil-based polymers. It provides a detailed examination of the classification, molecular structures, and synthetic routes of major bioplastics, including polylactic acid (PLA), polyglycolic acid (PGA), polycaprolactone (PCL), polybutylene succinate (PBS), polybutylene adipate-*co*-terephthalate (PBAT), and polyhydroxyalkanoates (PHAs). Special emphasis is placed on the unique properties and degradation behaviors of each material across various environmental conditions, such as industrial composting, soil, and marine ecosystems. The manuscript further discusses advanced strategies in polymer design, such as copolymerization, reactive blending, and incorporation of nano- or micro-scale additives, to enhance flexibility, thermal resistance, barrier properties, and mechanical integrity. In addition to technical advancements, the review critically addresses key limitations impeding large-scale commercialization, including high production costs, limited availability of bio-based monomers, and inadequate end-of-life treatment infrastructure. Finally, future research directions are proposed to advance the development of fully bio-based, functionally tunable, and circular bioplastics that meet the performance demands of modern applications while reducing environmental impact.

## 1. Introduction

**Figure 1:**
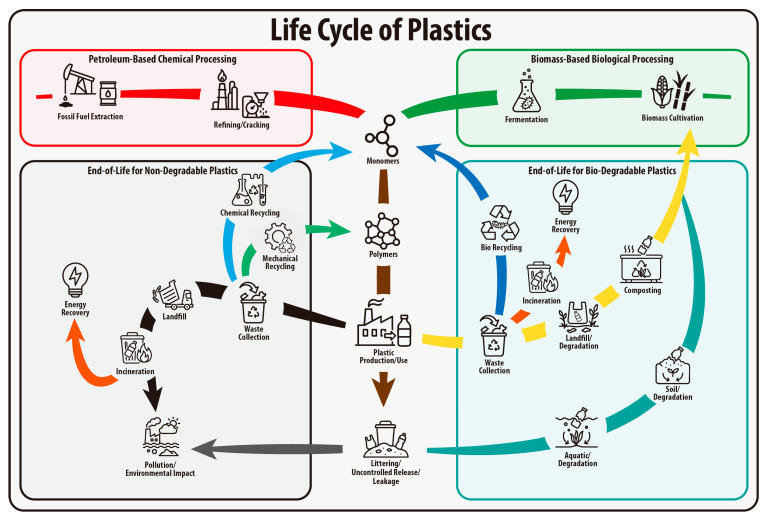
Schematic diagram of the life cycle of plastics from production to end-of-life (EOL).

Plastics are employed in various fields, ranging from common household objects to advanced materials for new technologies, due to their numerous advantages, including low cost, transparency, light-weight, processability, durability, and exceptional mechanical robustness [1]. The industrial production of plastics has significantly contributed to human convenience, but has also resulted in a continual rise in both their consumption and production. From 1950 to 2023, global plastic production grew consistently from 1.5 million tons to 413.8 million tons [2]. This rapid growth has resulted in approximately 6.3 billion tons of plastic waste by 2015, 78% of all plastics ever produced, of which only 9% was recycled, 12% incinerated, and 79% disposed of in landfills or the natural environment. If current trends continue, it is projected that by 2050, 12 billion tons of plastic waste will be either landfilled or littered into the environment [3]. This accumulation is particularly concerning because plastics can typically persist in landfills or the environment for hundreds to thousands of years due to their high resistance to degradation. Moreover, they break down into micro- and nanoplastics during weathering, potentially leading to further environmental issues. Microplastics (1 µm~5 mm) and nanoplastics (<1 µm) have the potential to disrupt ecosystems and may also pose risks to human health through circulation [4,5,6]. In response to the environmental challenges associated with traditional plastics, bioplastics have emerged as a promising alternative (Figure 1) [7].

**Figure 2:**
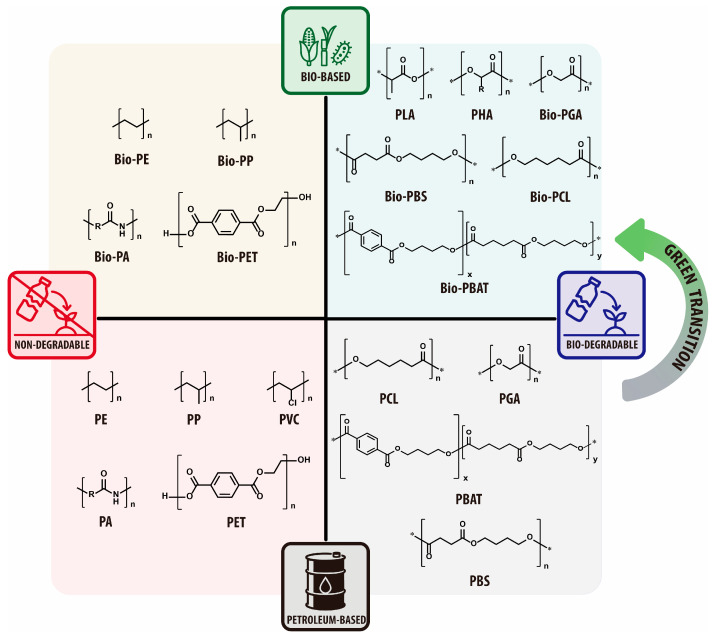
Classification of common plastics based on origin and biodegradability.

**Figure 3:**
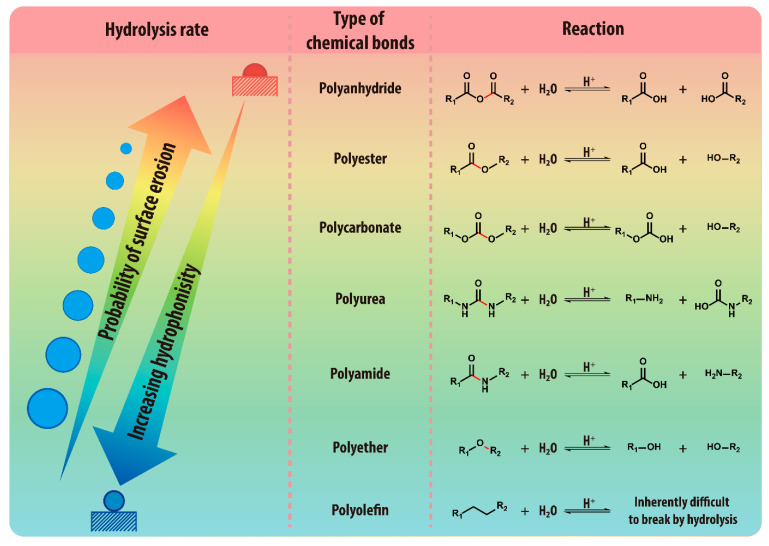
Relative hydrolysis rate of the main polymers susceptible to degradation upon exposure to humidity. Reproduced and modified from ref. [9] with permission from MDPI.

Bioplastics can be broadly categorized into two types based on their origin and biodegradability: bio-based bioplastics, which are produced from renewable resources such as corn and sugarcane, and biodegradable plastics, which can be broken down by microorganisms under specific conditions (Figure 2). It is important to note that bio-based bioplastics are not necessarily biodegradable, and some biodegradable plastics can be synthesized from petrochemical feedstocks [8]. Biodegradable plastics, mostly a subset of polyester, degrade through hydrolytic and/or microbial mechanisms under specific environmental conditions such as temperature, humidity, pH, and the presence of microorganisms (Figure 3) [9,10]. Unlike conventional plastics, which can remain in the environment for centuries, biodegradable plastics can break down into carbon dioxide (CO_2_), water (H_2_O), and methane (CH_4_) through microbial activity, reducing long-term waste when directed to appropriate end-of-life (EOL) pathways [11].

In addition to mitigating solid waste, biodegradable plastics, particularly those derived from renewable resources, can help reduce carbon emissions and dependence on fossil fuels. The use of biomass-based feedstocks can lower the overall carbon footprint of plastic production by partially or entirely replacing petroleum-based raw materials. During their life cycle, some bio-based biodegradable plastics may even offer net carbon savings through carbon capture and sequestration in the cultivation of renewable raw materials. For instance, Braskem (São Paulo, Brazil), the world’s largest polyolefin producer, reported that producing bio-polyethylene (Bio-PE) from sugarcane-derived ethylene can remove approximately 3.09 tons of CO_2_ from the atmosphere for every ton of Bio-PE produced [12]. Furthermore, by reducing dependency on petrochemical resources, bioplastics can contribute to a more sustainable and circular economy.

Despite their promise, biodegradable plastics face several critical limitations that restrict their widespread use and environmental effectiveness. In general, due to their lower strength, durability, and thermal stability compared to traditional plastics, biodegradable plastics are limited in applications requiring long-term performance or mechanical robustness. Furthermore, most biodegradable plastics require specific conditions, such as elevated temperatures (typically above 50 °C), regulated humidity, and active microbial populations, to degrade efficiently [13]. These conditions are primarily achieved in industrial composting facilities, which are not universally available. In natural environments like soil, rivers, or oceans, the absence of these optimized conditions results in significantly slower degradation rates, allowing biodegradable plastics to persist and potentially harm ecosystems much like conventional plastics. This discrepancy raises important concerns regarding their environmental effectiveness.

Moreover, biodegradable plastics present challenges to the current recycling infrastructure [14,15]. Since they are not compatible with traditional plastic recycling streams, their presence can contaminate recyclable materials, making recycling less cost-effective and reducing the quality of recycled products. Effective and appropriate sorting and separation systems are required to manage biodegradable plastics, but such systems are still underdeveloped in many regions. Furthermore, the introduction of various types of biodegradable plastics to conventional plastics significantly complicates recycling systems. Public awareness of these distinctions is still limited, increasing the risk of mis-sorting and contamination in recycling streams. Without adequate public education, collection infrastructure, and regulatory support, biodegradable plastics may end up in landfills or aquatic environments where their environmental advantages are significantly diminished.

Given these complexities, a comprehensive understanding of bioplastics is essential for assessing their potential as sustainable alternatives to conventional plastics. This review aims to provide a critical overview of biodegradable plastics, whether bio-based or petroleum-based, with a focus on their production methods, chemical structures, material properties, performance enhancement strategies, degradability, and environmental impacts. In addition, this review also provides a fundamental tutorial view of biodegradable plastics. By exploring current advancements, limitations, and future directions, this paper seeks to offer insights that can guide research, industry practices, and policy development toward a more sustainable plastic circular economy.

## 2. Biodegradable Bioplastics

### 2.1. Polylactic Acid (PLA)

#### 2.1.1. Overview and Synthesis

Polylactic acid (PLA) is a widely studied bio-based thermoplastic polyester, primarily produced from renewable resources such as corn starch, sugarcane, and other carbohydrate-rich agricultural byproducts [16]. Although often described as biodegradable, PLA degrades efficiently only under industrial composting conditions, while demonstrating much slower breakdown in natural environments. PLA currently holds the highest market share among bioplastics and represents the largest market size for a single bioplastic, resulting in its strong price competitiveness [17]. In contrast to many other biodegradable plastics, PLA distinctly provides excellent optical transparency with mechanical rigidity and strength, making it a promising alternative to traditional petroleum-based polymers [18]. In addition, PLA’s excellent thermal processability and low toxicity have enabled its use across a wide range of processing techniques and applications. Notably, PLA has become one of the most widely used filaments in the desktop 3D printer market, increasingly competing with ABS, which had traditionally dominated the field. Furthermore, owing to its origin from natural feedstocks and its ability to degrade under industrial composting conditions, PLA has emerged as a leading candidate for sustainable alternatives to fossil-based polymers.

**Figure 4:**
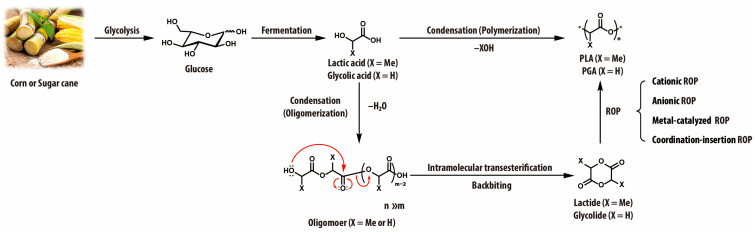
Synthetic pathways of PLA and PGA from renewable resources via step-growth condensation or chain-growth ring-opening polymerization (ROP).

The synthesis of PLA involves two major steps: monomer production (lactic acid or lactide) and polymerization (Figure 4) [19,20]. Monomer production begins with microbial fermentation of carbohydrates, primarily by *Lactobacillus* species, which convert glucose into lactic acid under controlled conditions [21]. To produce the alternative monomer, lactide, lactic acid undergoes oligomerization under reduced pressure, affording short-chain oligomers. These oligomers are subsequently cyclized into lactide via intramolecular transesterification (backbiting) in the presence of catalysts [22,23]. The polymerization proceeds via two main pathways: direct polycondensation, which follows a step-growth mechanism, and ring-opening polymerization (ROP), which proceeds through a chain-growth mechanism (Table 1). Direct polycondensation involves the thermal condensation of lactic acid under reduced pressure. However, it generally produces low-molecular-weight PLA due to the challenges associated with complete water removal from the reaction mixture [18,20]. It is worth noting that water or alcohols are the byproducts of the condensation reaction, and if not efficiently and continuously removed from the reaction mixture, they can drive the equilibrium backward, according to Le Châtelier’s principle, thereby limiting chain growth and preventing the formation of high-molecular-weight PLA. This fundamental principle applies not only to PLA but also to the synthesis of other polyesters formed through step-growth polymerization. To address this limitation, azeotropic polycondensation has been employed, using organic solvents to facilitate water removal, allowing higher molecular-weight PLAs [18]. ROP is the predominant approach for commercial PLA production, as it enables the synthesis of high-molecular-weight PLA in a precisely controlled manner using Lewis acid catalysts such as tin (II) octanoate (Sn(Oct)_2_). PLA prepared via ROP typically exhibits enhanced thermal stability and mechanical strength compared to PLA synthesized through polycondensation. The high efficiency, scalability, and tunability of this method have made ROP the preferred route for industrial PLA production, establishing PLA as a leading bioplastic for various commercial applications [19,20].

PLA exhibits a wide range of physical and mechanical properties that can be tailored through its stereochemistry. It is available in various stereoisomeric forms, poly(L-lactide) (PLLA), poly(D-lactide) (PDLA), and poly(DL-lactide) (PDLLA), which result from different stereochemical configurations of the lactic acid. These forms determine the crystallinity of the polymer, which in turn influences its mechanical and thermal behavior. PLLA and PDLA typically exhibit semi-crystalline structures and higher strength, whereas PDLLA is amorphous due to the random arrangement of L- and D-units [25]. PLA offers tensile strength (45 to 60 MPa) and elastic modulus (0.35 to 3.5 GPa) comparable to PET, and it also shows moderate thermal resistance with glass transition temperature (T_g_) typically between 45~65 °C and a melting temperature (T_m_) for crystalline PLLA ranging from 170 to 180 °C [22].

#### 2.1.2. Limitations and Recent Advances

Despite these promising properties, PLA has notable limitations. It is intrinsically brittle and typically exhibits an elongation at break of less than 10% [22]. In addition, thermal degradation occurs around 300 °C, which limits PLA’s thermal processing window [26]. Beyond the mechanical and processing limitations, PLA also suffers from restricted biodegradability. While it is compostable under industrial conditions and breaks down into CO_2_ and H_2_O within a few months, it demonstrates poor degradability in natural environments such as soil and marine ecosystems [27]. This is primarily due to its semi-crystalline structure and hydrophobic nature of PLA, which hinder water penetration and enzymatic activity. Additionally, incompatibility with traditional recycling systems and the relatively high production cost compared to petroleum-based plastics remain significant barriers to market expansion.

To address PLA’s inherent brittleness and low toughness, and limited biodegradability, extensive efforts have been devoted to blending strategies and copolymerization approaches. Blending PLA with poly(butylene adipate-*co*-terephthalate) (PBAT), for example, significantly improved ductility and toughness without compromising overall stiffness [28]. Incorporating 20 to 25 wt% of PBAT into PLA increased the elongation at break to approximately 300~350%, while maintaining higher Charpy impact strength than neat PLA. These enhancements were attributed to the fine dispersion of submicron-sized PBAT domains, which served as effective stress concentrators to dissipate impact energy. Similarly, blending PLA with 20 wt% poly(*ε*-caprolactone) (PCL) and 0.5 wt% lysine triisocyanate (LTI) resulted in a notched impact strength of 17.3 kJ/m^2^ and an elongation at break of 268%, highlighting the role of LTI as an effective compatibilizer in promoting interfacial adhesion and enhancing the mechanical properties of PLA/PCL blends [29]. In addition to the mechanical benefits, PLA/PCL blends also demonstrated significantly improved biodegradability. For example, a 20 wt% PCL blend achieved more than 90% biodegradation after 165 days under home-composting conditions (28 °C), and even 5 wt% PCL blend surpassed 90% biodegradation after 407 days, whereas neat PLA showed negligible degradation under the same conditions [30]. Moreover, in PLA/PBSA (poly(butylene succinate-*co*-adipate)) blends, the introduction of 0.6 wt% of a reactive compatibilizer (multifunctional epoxy chain extender) reduced the dispersed phase size from 2.69 μm to 0.7 μm, leading to an increase in impact strength to 38.4 kJ/m^2^ and an elongation at break of 179% [31]. In terms of biodegradability, PLA/PBSA (60/40) blends exhibited complete biodegradation after 210 days under home-composting conditions (28 °C). However, the biodegradation was markedly slower in soil (25 °C) and freshwater (21 °C) [30]. Nanofiller incorporation has also been employed to enhance PLA-based blend performance. For instance, in PLA/PBAT blends without C30B (organo-modified montmorillonite), the average dispersed PBAT phase size was approximately 1.3 μm; however, the addition of C30B significantly reduced the PBAT phase size to approximately 0.35 μm, which contributed to improved phase stability and mechanical performance [32]. Such compatibilization strategies effectively reduce interfacial tension, promote fine dispersion, and enhance stress transfer between phases, thereby contributing to substantial improvements in both impact strength and elongation at break.

Copolymerization, through the incorporation of co-monomers, is another powerful strategy to enhance the functional properties of PLA by introducing tailored flexibility, toughness, and biodegradability. For instance, poly(lactic acid-*co*-malic acid) (PLMA) copolymers synthesized via polycondensation of lactic acid and malic acid have shown significant mechanical enhancements when blended with PLA [33]. A PLA/PLMA (90/10 wt%) blend exhibited a remarkable increase in elongation at break from 4.2% (neat PLA) to 378.6%. More recently, poly(lactide-*co*-hydracrylate) (PLH), a copolymer of lactic acid and 3-hydroxypropionic acid, offers up to 20 times the flexibility of PLA while maintaining transparency [34]. Microbially produced poly(lactate-*co*-3-hydroxybutyrate) (PLAHB) copolymers also demonstrated substantial improvements in elongation behavior. PLA blended with 20 wt% P40LAHB, containing 40 mol% lactate, displayed an elongation at break exceeding 250% while maintaining a tensile modulus of 2.5 GPa, indicating enhanced toughness without significant compromise of mechanical integrity [35]. Beyond the enhancement of mechanical properties, PLA/P40LAHB blends (70/30) exhibited biodegradability of PLA in seawater. After more than one month of induction period, biodegradation initiated in the LAHB domains and subsequently progressed to PLA, ultimately confirming that PLA itself degraded within the blend in seawater conditions. In addition, poly(lactic acid-*co*-*ε*-caprolactone) (PLAC) copolymers provided improved mechanical strength and biodegradability, with successful demonstration in bladder tissue regeneration. Furthermore, amphiphilic block copolymers such as PLA-*b*-PEG have been extensively investigated for their ability to enhance the hydrophilicity and toughness of PLA, with potential applications in drug delivery and tissue engineering [36].

In parallel, crystallinity enhancement has proven essential for improving PLA’s heat resistance and dimensional stability. Thermal annealing of PLA at 120 °C for 2 h can increase crystallinity to ~44% and raise the heat deflection temperature (HDT) from 55 °C to 108 °C [37]. Stereocomplex PLA (sc-PLA), formed by equimolar blending of PLLA and PDLA, exhibits T_m_ around 220 °C and has been used to enhance the dimensional stability of PLA/PBAT blends [38]. Additionally, inorganic nucleating agents like talc can raise PLA’s crystallinity from 2% to 25%, increasing HDT to as high as 139 °C [39].

In summary, while PLA stands out as the most prominent bio-based and biodegradable thermoplastic due to its renewable origin, favorable mechanical properties, and industrial compostability, its practical limitations necessitate further innovation. Intrinsic brittleness, moderate thermal resistance, and limited degradability in natural environments remain critical challenges. Looking ahead, continued advancements in synthesis, structural modification, and EOL management will be critical to establishing PLA as a truly sustainable alternative. As the bioplastics industry evolves, PLA remains a central material, offering valuable insights into the challenges and opportunities of replacing conventional plastics with renewable, biodegradable counterparts.

### 2.2. Polyglycolic Acid (PGA)

#### 2.2.1. Overview and Synthesis

Polyglycolic acid (PGA) is the simplest aliphatic polyester and a widely studied biodegradable polymer, known for its high crystallinity and rapid hydrolytic degradability [40,41]. It belongs to the poly(*α*-hydroxy acid) family (alongside PLA) and consists of repeating glycolate units without pendant side groups, allowing tight chain packing [40]. The polymer is biocompatible and bioresorbable, producing only non-toxic glycolic acid upon degradation [41]. These features have made PGA invaluable in biomedical applications such as sutures, tissue engineering, drug delivery systems (DDS), and temporary implants [42]. More recently, its excellent gas barrier properties and compostability have led to increased investigation of PGA in food packaging films and other industrial applications [43] (Table 2).

PGA is primarily synthesized via ROP of glycolide, a six-membered cyclic dimer of glycolic acid (Figure 4). This method enables high molecular weights (*M*_n_ up to 10^5^~10^6^ g/mol) with Lewis acid catalysts such as tin or bismuth-based compounds. Alternatively, direct polycondensation of glycolic acid is more cost-effective but typically yields low-molecular-weight PGA due to water removal challenges and equilibrium limitations [42]. To obtain higher molecular weights, high vacuum or azeotropic techniques are required to facilitate water removal [44]. Nevertheless, classical melt or solution polycondensation typically yields only oligomers or low-molecular-weight PGA, as the reaction rate decreases with increasing viscosity [45]. To address these limitations, multi-step processes have been utilized. One effective method involves initial melt condensation of glycolic acid, followed by solid-state polymerization at elevated temperatures (e.g., ~230 °C), allowing further chain extension [46]. Azeotropic polycondensation using suitable solvent systems has also been employed to improve molecular weight control, producing PGA with higher molecular weights, higher solubility, and faster degradation compared to conventionally prepared materials [41].

PGA, derived from glycolic acid, lacks the methyl side group present in PLA, which contributes to its greater crystallinity and mechanical properties [40]. PGA exhibits typically 45~55% crystallinity and demonstrates high tensile strength and stiffness, making it one of the strongest biodegradable polyesters. Oriented PGA fibers and films typically achieve tensile strengths around 115 MPa and Young’s moduli of approximately 7 GPa. These values significantly exceed those of PLA under comparable conditions, reflecting the tightly packed crystalline domains of PGA [18,40]. Despite the high crystallinity of PGA, it demonstrates a relatively low T_g_ of around 40 °C, indicating a transition to a rubbery state near body temperature [18,47]. It is important to note that the low T_g_ of PGA can be attributed to its chemical structure, specifically the lower steric hindrance for internal chain rotation. This facilitates efficient chain packing in the crystalline regions while simultaneously allowing greater chain mobility in the amorphous regions, resulting in its thermal softness below the melting point. Moreover, owing to the absence of the methyl side group found in PLA, PGA is more hydrophilic and thus undergoes rapid biodegradation primarily through hydrolysis of its ester bonds without requiring enzymatic activity [48]. As a result, PGA undergoes bulk erosion at a relatively fast rate. Complete degradation of PGA can occur within a few weeks to a few months, depending on the material’s size, form, and crystallinity [42]. Thin sutures are often resorbed in 6–8 weeks, while larger implants may require several months. PGA’s tunable degradation profile has been a major advantage in biomedical applications requiring temporary structural support.

#### 2.2.2. Limitations and Recent Advances

Despite its desirable properties, PGA presents several inherent limitations that complicate manufacturing and practical applications. A major limitation of PGA lies in its inherent mechanical brittleness, originating from its highly crystalline structure and lack of flexible chain segments. Unmodified PGA has low ductility, with elongation at break typically around 5~15%, making it prone to cracking under stress [49,50]. In contrast to its relatively low T_g_, PGA exhibits a high T_m_ of approximately 220~230 °C, indicative of strong interactions in the crystalline regions [18,40]. Although the wide thermal gap between T_g_ and T_m_ provides stability in the solid state, PGA’s melt processing is challenging due to the proximity of its melting temperature to its thermal degradation, which begins around 280~300 °C [51]. This narrow margin demands precise temperature control during processing to prevent premature polymer degradation. Moreover, PGA’s hydrophilicity presents additional challenges during processing and storage. Due to its high sensitivity to moisture, thorough drying is essential before melt processing, as residual water can trigger hydrolytic degradation at elevated temperatures [50]. Even under ambient conditions, PGA is susceptible to gradual hydrolysis unless it is stored in well-controlled, low-humidity environments [47,49].

To address the intrinsic brittleness and rapid hydrolysis of PGA, copolymerization has been widely adopted as a strategy to modulate its mechanical flexibility and degradation behavior. Among various approaches, random copolymerization with lactide has been extensively studied. PLGA (poly(lactic acid-*co*-glycolic acid) copolymer containing 50 mol% lactic acid displayed improved ductility compared to neat PGA, with an elongation at break of approximately 38% [52]. Moreover, it undergoes complete hydrolytic degradation within approximately 1~2 months. The flexibility of PGA can be further improved by incorporating more flexible comonomers like *ε*-caprolactone, although it compromises mechanical robustness [53]. Alternatively, block copolymers represent a strategy for balancing mechanical properties and degradation behavior. For instance, a triblock PGA-PTMC-PGA copolymer (PTMC: poly(trimethylene carbonate)) showed much less elasticity than its random copolymer but exhibited a much slower hydrolysis rate, highlighting the trade-off between mechanical toughness and degradation speed governed by the microstructure of a polymer [54]. Recent advances in regioselective ROP have enabled the synthesis of sequence-controlled polyesters. One notable example is the alternating copolymer of glycolic acid and mandelic acid, where the rigid aromatic units impart elevated T_g_ up to 91.3 °C along with thermal and mechanical stability relative to conventional PGA [55].

In addition to copolymerization, blending PGA with other biodegradable polymers has been employed to improve toughness and modulate its hydrolytic degradation rate [56,57,58]. However, neat PGA is typically immiscible with many biodegradable polymers, leading to phase separation that undermines mechanical performance [56,59]. Compatibilizers or chain extenders are, therefore, added to promote interfacial adhesion in PGA blends. Conventional compatibilizers, such as multi-functional epoxides or isocyanate-based chain extenders, have proven effective in toughening PGA blends, but they are typically petroleum-derived, expensive, and non-degradable [56,58,60]. This has prompted the development of bio-based, degradable additives as sustainable alternatives. Notably, epoxidized soybean oil (ESO) has gained attention as a low-cost, fully bio-based reactive compatibilizer. For instance, a composite additive combining ESO with tannic acid was introduced to PGA/PCL (90/10) blends, and the addition of 1~5 wt% of the ESO-tannic acid compatibilizer increased the elongation at break from 5.5% (neat PGA) to 118~150% [61]. In addition to improving ductility, the compatibilizer moderated the hydrolysis rate of PGA, slowing down the degradation by approximately 22~25%. In a similar example, incorporation of a small amount (0.7 phr) of a branched ESO-based compatibilizer in PGA/poly (butylene succinate) (PBS) blend significantly refined phase morphology, reducing the dispersed domain size from ~2.04 µm to ~0.45 µm [60]. This refined microstructure translated into significantly improved mechanical properties.

Overall, PGA has emerged as a sustainable polymer with growing potential across diverse applications. Its rapid biodegradability, high mechanical strength, and excellent gas barrier properties make it particularly attractive for eco-friendly packaging. In parallel, its biocompatibility has enabled widespread biomedical applications. Recent progress in copolymer design, reactive compatibilization, and degradation control has significantly extended the application potential of PGA. Chemical modifications such as flexible comonomer incorporation or sequence control can tailor mechanical properties and adjust degradation kinetics to meet specific application needs. Continued research into these molecular and processing strategies is expected to further broaden PGA’s applicability, reinforcing its position as a versatile and sustainable material.

### 2.3. Polycaprolactone (PCL)

#### 2.3.1. Overview and Synthesis

Polycaprolactone (PCL) is a biodegradable aliphatic polyester mainly produced from fossil-derived *ε*-caprolactone. Despite not being bio-based, PCL gained significant attention for its adjustable biodegradability, long-term biocompatibility, and mechanical flexibility, making it a versatile material in biomedical and environmental applications [62,63]. PCL exhibited a flexible nature with a relatively low melting point and glass transition temperature, facilitating easy thermal processing through conventional methods. These properties, combined with its low toxicity, have led to widespread use of PCL in medical applications, including sutures, DDS, and long-term biodegradable implants. Notably, PCL has been approved by the U.S. Food and Drug Administration (FDA) for specific biomedical applications, reinforcing its clinical relevance and safety profile. In addition to its biomedical use, PCL has attracted growing interest in compostable materials due to its environmental degradability and compatibility with other biopolymers. Its ability to blend with other biodegradable polymers and potential for chemical modification further enhance its versatility across a broad range of applications. These combined attributes position PCL as a key material for advanced, functionally degradable polymers for both biomedical and environmental fields.

The synthesis of PCL follows principles similar to those used in PLA and PGA production, involving either step-growth polycondensation or chain-growth ROP, with the latter being the predominant route for high molecular weight PCL. ROP of ε-caprolactone using Lewis acid catalysts such as aluminum isopropoxide (Al(O-*i*-Pr)_3_) and (Sn(Oct)_2_) typically yields PCL with number-average molecular weights up to approximately ~90,000 g/mol, with a narrow polydispersity index (PDI). Higher molecular weights exceeding 130,000 g/mol have been reported when using alkali metal catalysts such as sodium cyclopentadienide [63]. Alternatively, enzymatic catalysts such as *Candida antarctica* lipase offer a green, solvent-free route, yielding PCL with *M*_n_ values in the range of 14,900–35,600 g/mol and PDI below 1.72 [64]. More recently, organic catalysts, including organic acids (tartaric acid or methanesulfonic acid, etc.) and bases (1,5,7-triazabicyclo[4.4.0]dec-5-ene (TBD) or phosphazene base, etc.), have enabled low-temperature polymerization (0~50 °C), which enhances control over molecular weight distribution and minimizes undesirable side reactions [63].

PCL exhibits a unique combination of material properties. As a semi-crystalline aliphatic polyester with a relatively low T_m_ of approximately 60 °C and T_g_ around −60 °C, it offers excellent flexibility and thermal processability [65,66]. Its semi-crystalline nature, with a crystallinity typically ranging from 45% to 70%, significantly influences its mechanical strength, thermal behavior, and hydrolytic degradation rate [65,67]. PCL shows structural stability under physiological conditions and degrade slowly over 2 to 4 years without generating harmful acidic byproducts, making them appropriate for long-term biomedical implants such as tissue-engineering and drug delivery [68,69]. Furthermore, they have also demonstrated beneficial effects in skin regeneration and wound healing due to their biodegradability and mechanical compliance [70]. Beyond the medical applications, PCL’s versatility extends to environmental applications, including biodegradable packaging and agricultural mulching films, thanks to its ability to degrade under composting conditions without leaving toxic residues [71]. Its broad compatibility with other materials and suitability for various processing techniques further emphasize PCL’s potential as a sustainable alternative to traditional, non-degradable polymers across multiple fields.

#### 2.3.2. Limitations and Recent Advances

Despite these advantages, the practical application of PCL is constrained by its inherent immiscibility with other polymers, particularly PLA. In PLA/PCL blends without compatibilizers, this incompatibility leads to poor interfacial adhesion and phase-separated morphologies, which compromise mechanical integrity. For example, PLA/PCL (80/20 wt%) demonstrated that Young’s modulus is reduced from 3.2 GPa (neat PLA) to 2.8 GPa, the average domain size of dispersed PCL increased from 0.31 µm to 0.76 µm, indicating pronounced coalescence and diminished mechanical stability [72].

These limitations restrict the performance of PCL in composite applications and highlight the need for further research into compatibilization, copolymerization, and functionalization strategies to expand its utility in advanced functional materials. Various compatibilization strategies have been developed to tailor the phase morphology and improve mechanical properties. One effective approach utilizes amphiphilic surfactants such as Pluronic (poloxamer), which reduce interfacial tension between PLA and PCL phases [73]. The addition of 2.5 phr Pluronic resulted in a finer dispersion of PCL domains and a substantial improvement in elongation at break and toughness, which can be attributed to the improved stress transfer across the interface. Bio-based compatibilizers with multifunctionality, such as polyamide-grafted bamboo charcoal (PA-*g*-BC), have also been explored [74]. In PLA/PCL (80/20) blends, the incorporation of PA-*g*-BC improved tensile strength to 18 MPa and elongation at break to 223%, while also raising the limiting oxygen index (LOI) to 30.8%, indicating enhanced flame retardancy alongside mechanical reinforcement. Further developments have focused on incorporating nanocellulose to enhance the crystallization and mechanical performance of PCL. The addition of nanocellulose (10 wt%) significantly increased the crystallization peak temperature from 18.8 °C (neat PCL) to 30.9 °C and reduced the isothermal crystallization half-time at 40 °C from 12.2 to 2.0 min [75]. This improvement is attributed to nanocellulose acting as an effective nucleating agent, improving crystallization kinetics without altering PCL’s crystal structure. This also led to increased stiffness and strength, though at the cost of reduced elongation at break, highlighting a trade-off between mechanical strength and ductility.

**Figure 5:**
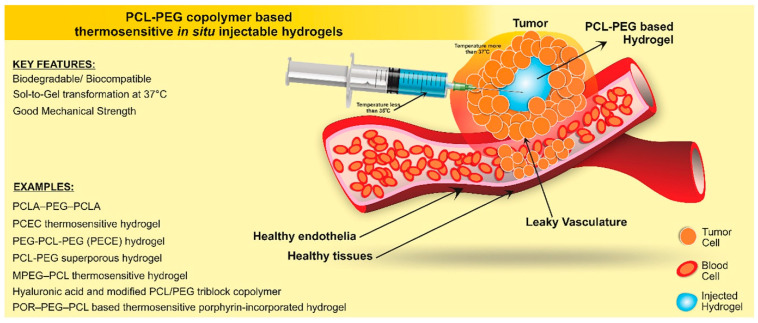
PCL-PEG copolymer-based thermosensitive injectable hydrogel. Reproduced from ref. [76] with permission from Elsevier.

Recent advances in side-chain functionalization and block copolymer design have significantly enhanced the biomedical potential of PCL, particularly in DDS [76,77,78,79]. Amphiphilic block copolymers combining PCL with PEG have been extensively developed for their ability to self-assemble into micelles or hydrogels that encapsulate hydrophobic drugs and enable controlled release (Figure 5). For example, PEG-*b*-PCL-based nanocarriers have shown improved solubility and intracellular delivery of paclitaxel, while star-shaped PCL/PEG micelles loaded with doxorubicin demonstrated enhanced anticancer activity in colon cancer models [79]. Similarly, PCL-PEG-PCL triblock copolymers exhibit temperature-sensitive sol–gel transitions near physiological conditions, forming injectable hydrogels suitable for sustained drug delivery and wound healing applications [76]. Drug-conjugated micellar systems also offer promising platforms for targeted therapy. For instance, methotrexate-conjugated mPEG-PCL diblock copolymers enabled pH-sensitive release with enhanced therapeutic efficacy and minimized undesirable drug leakage [80]. Another approach involves functionalizing PCL side chains with γ-valproate ester moieties, enabling self-assembled micelles that gradually release histone deacetylase (HDAC) inhibitors such as valproic acid (VPA), with biodegradation observed under mildly acidic conditions [81]. These formulations highlight how molecular engineering can precisely tune pharmacokinetics and cellular uptake.

Parallel to advances in DDS, strategies have emerged to address PCL’s inherent limitations, such as its slow degradation rate and hydrophobicity. One major approach is to incorporate hydrophilic and biodegradable segments into the polymer backbone. For example, PEG-PCL-PEG hydrogels not only support long-term stability but also accelerate degradation due to increased hydrophilicity and segmental flexibility [81]. Notably, polysaccharide-grafted PCL copolymers, including chitosan and agarose derivatives, have demonstrated enhanced biocompatibility, prolonged circulation, and stimulus-responsive release [71]. These systems offer a bridge between controlled biodegradation and biofunctionality, making them promising candidates for next-generation sustainable DDS.

Advances in blend optimization and copolymer design have significantly expanded the functional scope of PCL. Compatibilization strategies have improved phase morphology and mechanical integrity in PLA/PCL blends, while structurally engineered copolymers have addressed key limitations, including slow degradation, hydrophobicity, and limited biofunctionality. Through precise control of macromolecular architecture and composition, PCL-based materials have been tailored for diverse biomedical uses. However, despite these molecular-level improvements, the EOL management of PCL remains underdeveloped. Its relatively low melting point hinders compatibility with conventional thermal recycling processes, while its incompatibility with existing plastic sorting infrastructures represents a significant barrier to circular material flows. To meet broader sustainability objectives, future research should prioritize the development of bio-based monomeric alternatives, the implementation of strategies that focus on design for recycling, and the formulation of materials compatible with current waste management systems, thereby ensuring both high functional performance and life cycle circularity.

### 2.4. Poly(Butylene Succinate) (PBS)

#### 2.4.1. Overview and Synthesis

Poly(butylene succinate) (PBS) is a biodegradable aliphatic polyester prepared by melt polycondensation of 1,4-butanediol (BDO) and succinic acid (SA), both of which are accessible from either petroleum-derived or renewable bio-based sources, such as corn, cassava, and sugarcane [82,83]. PBS is a linear, semi-crystalline polymer that exhibits a favorable balance of strength and flexibility, with mechanical properties comparable to those of LDPE and PP. In addition, it offers excellent thermoplastic processability, mechanical strength, versatility in industrial fabrication, and moderate biodegradability, making PBS a promising sustainable alternative for applications including packaging, agricultural films, and disposable products [83]. Importantly, PBS and its blends have also shown promise in marine-related applications such as fishing nets and gears, where their mechanical robustness and partial degradability help reduce the environmental burden from ghost fishing gear [84].

The synthesis of PBS typically follows a step-growth polymerization mechanism, which consists of two key stages: an initial esterification reaction between succinic acid (or its dimethyl/diethyl ester) and BDO to form oligomeric intermediates, followed by a vacuum-assisted polycondensation step that drives the formation of high-molecular-weight polymers through the continuous removal of water or alcohol byproducts [83]. As a diacid–diol-based polyester, PBS is inherently synthesized as an alternating copolymer via step-growth polymerization. It is worth noting that while chain-growth ROP of PBS using a cyclic butylene succinate monomer has been demonstrated, this approach remains largely impractical for large-scale production due to the limited availability, complex synthesis of the required cyclic monomers [85]. Consequently, melt polycondensation remains the dominant and commercially viable method for PBS synthesis. Within this route, the use of metal-based catalysts, such as Ti(OBu)_4_ or SnCl_2_, has enabled the production of PBS with moderate to high molecular weights [82].

PBS possesses a semi-crystalline structure that gives advantageous mechanical and thermal properties for practical uses. It typically exhibits a crystallinity of 30~50%, with the α-phase being the most thermodynamically stable configuration [86]. The high crystallinity enhances its tensile strength and modulus, making PBS appropriate for use in packaging films, agricultural mulch, and biomedical devices. Surprisingly, PBS is highly ductile even with high crystallinity, exhibiting an elongation at break often near 230% and an elastic modulus around 700 MPa, depending on the degree of crystallinity. In addition, PBS provides an adequate processing window, with T_g_ of approximately −30 °C and T_m_ near 120 °C [82].

#### 2.4.2. Limitations and Recent Advances

From an environmental standpoint, PBS is recognized as an industrially compostable polyester and shows biodegradability under aerobic conditions. However, its degradation in natural environments such as soil and marine ecosystems proceeds at a much slower rate than in controlled composting facilities [84,87]. This limited degradability is primarily due to its high crystallinity and hydrophobicity, which hinder water diffusion and consequently inhibit complete biodegradation under ambient conditions. For example, ^13^C-labeled PBS cultured in soil for 425 days attained merely 65% mineralization to CO_2_ [87]. In addition, a recent study systematically evaluated the degradation behavior of PBS monofilaments under simulated marine sediment conditions, demonstrating only 27.3% mineralization to CO_2_ over 180 days, highlighting the ongoing challenge of complete degradation [84]. These structural constraints have driven the development of material design strategies to enhance the environmental performance and mechanical robustness of PBS. Recent efforts have focused on blending and compatibilization techniques to fine-tune the mechanical, thermal, and biodegradable properties of PBS [83]. In parallel, copolymerization with flexible or hydrophilic components has been employed to reduce crystallinity, improve water uptake, and accelerate both hydrolytic and microbial degradation [86,88].

Blending with both natural and synthetic polymers has yielded notable property enhancements. For instance, incorporating 50 wt% recycled cellulose into PBS increased the Young’s modulus over 700 MPa [89]. In addition, using branching agents such as glycerol propoxylate led to simultaneous improvements in tensile strength (up to 41 MPa) and elongation at break (>300%), attributed to the formation of long-chain and hyperbranched architectures that facilitate efficient energy dissipation under stress [90]. Reactive compatibilization has further improved the performance of PBS blends. In a PBS/poly(3-hydroxybutyrate-*co*-3-hydroxyvalerate) (PHBV) system, the addition of dicumyl peroxide (DCP) resulted in synergistic improvements in both tensile strength and elongation while retaining biodegradability [91]. Reinforcement with natural fibers further broadens the performance of PBS. Incorporation of 30 wt% kenaf fiber has been shown to increase the tensile modulus by 53% (to 664 MPa) and the storage modulus at 40 °C by 154% (to 1.70 GPa), while also accelerating crystallization and reducing the half-time of isothermal crystallization by up to 89.2% [92].

Complementary to these blending approaches, copolymerization provides a versatile route for modulating PBS properties at the molecular level. Copolymers incorporating functional comonomers such as butylene fumarate, *ε*-caprolactone, butylene adipate, furandicarboxylate, and salicylic acid have expanded thermal, mechanical, and biodegradation profiles of PBS. For example, random copolymers of poly(butylene succinate-*co*-butylene fumarate) (PBSF), synthesized via direct melt polycondensation, displayed linear increases in T_m_ and crystallization temperature (T_c_) with increasing butylene fumarate (BF) content due to isomorphic co-crystallization. PBSF30 (30 mol% BF), for instance, exhibited a T_m_ of ~120 °C and a modulus of 393 MPa, though its elongation at break remained less than 2% [93]. Blending of PBSF into PBS has also demonstrated a nucleation effect, as the addition of 2.0 wt% PBSF50 yielded even higher nucleation efficiency [94]. To improve mechanical balance, multiblock copolymers such as PBF-*b*-PBS were prepared to form coherent cocrystals and achieve a more favorable performance, with reported elongation at break of 156% and tensile strength of 45 MPa [95]. Crosslinked networks of PBS-*b*-PBF through internal double bonds of BF by UV curing exhibited tunable stiffness and degradation rates, offering design flexibility for specific performance targets [93].

Beyond these examples, various copolymer systems have demonstrated unique functional benefits. Block copolymers incorporating poly(tetramethylene oxide) improved toughness through enhanced segmental mobility while maintaining phase-separated crystallization [96]. PBS-*co*-PCL copolymers exhibited isodimorphic crystallization behavior, where increasing CL content decreases crystallinity and T_m_ from ~114 °C to ~40 °C [97]. A multiblock PBS/PCL (70/30) copolymer showed elongation at break over 700% and exhibited dual melting points at ~55 °C and ~110 °C, emphasizing the advantage of phase-separated morphology [98]. In addition, copolymers with salicylic acid showed improved enzymatic degradability, suitable for biomedical and agricultural applications [99]. Similarly, incorporation of triethylene succinate has been shown to reduce crystallinity and accelerate hydrolytic degradation [86].

Thanks to these advancements, PBS is now utilized across diverse sectors as packaging, agriculture, and insulation. Its thermal resistance and biodegradability make it ideal for biodegradable films, food trays, and mulching sheets. In agriculture, PBS-based fertilizer coatings and mulching films promote in-soil degradation and controlled nutrient release, minimizing environmental impact [100,101,102,103]. PBS foams are also valued in insulation and transport applications owing to their low thermal conductivity and cushioning properties. One emerging application is in fishing gear and marine equipment, where conventional plastic products, particularly nylon, are often lost or abandoned, contributing to ghost fishing and persistent plastic pollution in marine ecosystems [84]. While the mechanical robustness and flexibility of PBS make it a promising alternative for such uses, its slow degradation in cold, oxygen-limited marine environments presents a significant challenge. Nonetheless, its tunable degradation and synthetic versatility provide a valuable platform for developing next-generation marine-degradable polymers aimed at mitigating ocean plastic pollution [83].

Overall, ongoing innovations in PBS molecular design and processing strategies are expected to expand its functional capabilities and strengthen its position as a sustainable material. Future research focusing on molecular dynamics, crystallization kinetics, and copolymerization pathways may unlock new properties and enable broader implementation in advanced applications.

### 2.5. Poly(Butylene Adipate-co-Terephthalate) (PBAT)

#### 2.5.1. Overview and Synthesis

**Figure 6:**
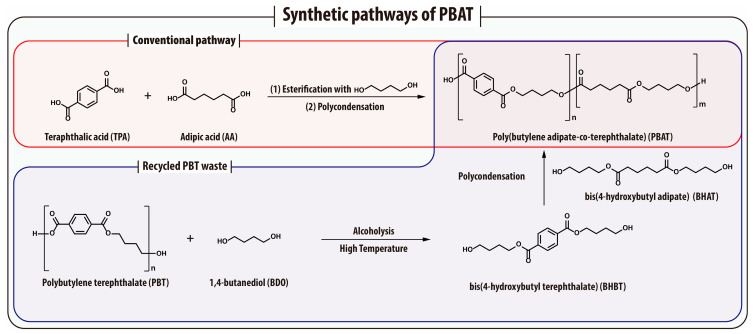
Synthetic pathways of PBAT from petrochemical feedstocks and recycled PBT waste.

Poly(butylene adipate-*co*-terephthalate) (PBAT) is a synthetic, biodegradable aliphatic-aromatic copolyester typically synthesized from fossil-derived monomers, including BDO, adipic acid (AA), and terephthalic acid (TPA) [104,105]. PBAT can be viewed as a random copolymer of polybutylene terephthalate (PBT) and polybutylene adipate (PBA), consisting of flexible aliphatic segments (AA and BDO) and rigid aromatic segments (TPA) (Figure 6). This molecular architecture imparts PBAT with a unique balance of flexibility and mechanical robustness, making it suitable for applications that demand both ductility and strength [106]. While aliphatic polyesters are generally known for their high flexibility, PBAT achieves comparable elongation at break, typically between 600~800%, and also benefits from enhanced thermal and mechanical properties due to the presence of aromatic units [105,107]. Its performance characteristics resemble those of LDPE, particularly in terms of processability and printability, making PBAT a promising alternative to conventional petroleum-based plastics.

Although PBAT was originally developed and remains largely from fossil-derived monomers, recent advances are gradually shifting its production toward more sustainable alternatives. BDO and AA can now be obtained from renewable sources through microbial fermentation, allowing for the production of bio-based PBAT with a biomass content of up to ~68% [108]. However, achieving 100% biomass-derived PBAT remains challenging due to the absence of an economically viable and scalable bio-based route or catalytic conversion for TPA. TPA is traditionally produced from petrochemical *p*-xylene, and while bio-based alternatives such as 2,5-furandicarboxylic acid (FDCA) or bio-*p*-xylene have been proposed, they face several obstacles, including complex synthesis pathways and high production costs [109,110]. As a result, despite progress in replacing some monomers with renewable counterparts, the full transition to completely bio-based PBAT is still limited by the technological and economic bottlenecks associated with bio-TPA production. Nonetheless, growing interest in renewable alternatives has led to increased research efforts focused on improving the environmental profile of PBAT. In addition to its biodegradability, PBAT exhibits excellent film-forming ability, thermal processability, and high flexibility, supporting its widespread use in compostable bags, flexible food packaging, agricultural mulch films, and disposable hygiene products [104,111]. Its enzymatic hydrolyzability and proven compostability under industrial conditions further reinforce its role as a high-performance, functional biodegradable material [112].

PBAT is commonly synthesized via a two-step melt polycondensation process involving esterification (or transesterification) followed by high-vacuum polycondensation of BDO, AA, and TPA (or their ester derivatives) [104,105]. In the initial step, the diol and dicarboxylic acid monomers undergo esterification to form oligomers, which are subsequently polymerized at elevated temperatures under reduced pressure to afford high-molecular-weight PBAT [104,105,108]. This process is fully compatible with existing polyester manufacturing infrastructure and allows for large-scale production [104,106]. However, unlike some aliphatic polyesters such as PLA, PGA, or PCL, PBAT cannot be readily synthesized via chain-growth ROP, due to the absence of suitable cyclic ester precursors for its co-monomers. This structural limitation inherently constrains the synthesis of PBAT to step-growth polymerization, making it more difficult to precisely control molecular weight and dispersity. Catalysts play a pivotal role in both oligomerization and polymerization. Titanium-based catalysts, particularly titanium alkoxides, are widely utilized due to their high thermal stability and catalytic efficiency [113]. In addition, tin, antimony, and iron-based compounds, including their oxides, alkoxides, and acetates, have been employed to accelerate reaction rates and improve polymer quality by minimizing side reactions and thermal degradation [113]. In addition to conventional synthesis, a chemical recycling approach has been proposed using polybutylene terephthalate (PBT) as an alternative feedstock. Alcoholysis of PBT with BDO produces bis(4-hydroxybutyl terephthalate) (BHBT), which is subsequently polymerized with bis(4-hydroxybutyl adipate) (BHAT), synthesized from adipic acid and BDO, to yield PBAT. This approach not only enables high-purity PBAT production but also supports the circular polymer economy through the incorporation of recycled polyester waste (Figure 6) [114].

#### 2.5.2. Limitations and Recent Advances

Despite its advantages, PBAT exhibits moderate tensile strength and modulus, which limits its utility in load-bearing applications. To address these limitations, reactive blending and compatibilization strategies have been explored to enhance the mechanical performance of PBAT. For example, an 80/20 PBAT/PLA blend modified with epoxy-functionalized acrylic polymer (Joncryl ADR^®^-4368) showed an increase in tensile strength from 22 MPa to 38 MPa, representing a 72% enhancement compared to the unmodified blend. Although the elongation at break decreased from 949% (neat PBAT) to 579%, phase compatibility and overall mechanical stability were improved [115]. Blending PBAT with plasticized starch using maleated PBAT (PBAT-*g*-MA) as a compatibilizer further improved mechanical strength and flexibility through enhanced interfacial adhesion and domain size reduction [116].

In addition to its mechanical limitations, PBAT also suffers from poor gas barrier properties, which restrict its use in packaging applications requiring high moisture or oxygen resistance. To address this drawback, various formulation strategies incorporating barrier-enhancing additives have been developed. For instance, a PBAT/PGA blend film incorporating 15 wt% PGA and an antioxidant system achieved a tensile strength of 48.6 MPa while reducing the water vapor transmission rate by 60%, from 15.9 × 10^−14^ to 6.3 × 10^−14^ g·cm/(cm^2^·s·Pa) [117]. In another study, a PBAT composite film incorporating 4 wt% cellulose nanofiber grafted with PHBV chains exhibited a 44% reduction in oxygen permeability and a 32% reduction in water vapor permeability compared to neat PBAT. These enhancements were attributed to the hydrophobic nature of PHBV, improved interfacial adhesion between filler and matrix, and increased crystallinity, which together extended the diffusion pathway for permeating molecules [118]. Additionally, multilayer PBAT-based nanocomposite coating films with a thin layer of sulfonated PBAT and exfoliated sodium montmorillonite (Na-MMT) achieved outstanding oxygen barrier performance [119]. Oxygen permeability values as low as 0.0016 barrer were reported, representing up to a 631-fold reduction compared to neat PBAT. Mechanical properties were well maintained, with tensile strength from 15~20 MPa and elongation at break between 600~730% [120].

Another limitation of PBAT lies in its slow degradation in natural environments, primarily due to π–π stacking of the rigid segments originating from TPA. Moreover, the release of TPA as a degradation byproduct raises environmental toxicity concerns. To further improve environmental performance and reduce reliance on fossil-derived aromatics, FDCA has been explored as a promising bio-based alternative to TPA. FDCA-based copolyesters, such as poly(butylene adipate-*co*-furanoate) (PBAF), display enhanced enzymatic hydrolysis owing to the increased polarity and flexibility of the furan ring, promoting hydrolysis of adjacent ester bonds [116]. This selective degradation has been verified by FT-IR and two-dimensional correlation spectroscopy. Additional gains have been achieved by incorporating diglycolic acid (DGA) into copolyesters like poly(butylene diglycolate-*co*-furandicarboxylate) (PBDF) improves hydrophilicity and enzymatic degradability relative to PBAT [121].

Looking ahead, ongoing advancements in copolymer design, blend optimization, and nanocomposite strategies are progressively enhancing its performance and environmental compatibility. Advances in monomer sourcing, particularly efforts to develop cost-effective bio-based pathways for BDO, AA, and TPA alternatives, are central to improve the material’s sustainability. In addition, structural modifications and functional blending strategies are expanding its mechanical versatility and application scope. Future research targeting enzymatic degradation mechanisms, interfacial interactions in blends and composites, and scalable bio-based monomer synthesis is expected to further elevate PBAT’s potential as a next-generation biodegradable polymer. With integrated progress in both material design and circular EOL strategies, PBAT stands to play a pivotal role in the transition toward a sustainable plastic economy.

### 2.6. Polyhydroxyalkanoates (PHAs)

#### 2.6.1. Overview and Synthesis

**Figure 7:**
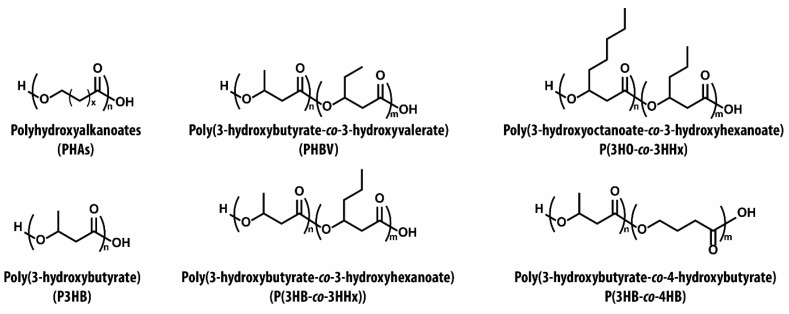
Chemical structures of various polyhydroxyalkanoates (PHAs).

Polyhydroxyalkanoates (PHAs), linear polyesters composed of hydroxyalkanoate monomers connected through ester linkages, represent a diverse and promising class of bio-based, biodegradable polyesters produced by microbial fermentation of renewable feedstocks such as sugars, fatty acids, glycerol, and agricultural residues (Figure 7). Unlike chemically polymerized bio-based or biodegradable polymers, PHAs are biosynthesized directly by microorganisms under nutrient-limited conditions in the presence of excess carbon sources [122,123]. These intracellular carbon and energy storage polymers are accumulated as granules by various microbial strains such as *Cupriavidus necator*, *Ralstonia eutropha*, *Pseudomonas* spp., *Bacillus* spp., and *Halomonas* spp. [124,125,126,127]. Due to their entirely biological origin, PHAs are universally regarded as 100% bio-based and biodegradable, positioning them uniquely among sustainable polymers. The properties of PHAs vary significantly depending on chain length and composition, allowing for a broad range of tunable physical and mechanical characteristics. PHAs are typically classified as short-chain-length PHAs (scl-PHAs), such as poly(3-hydroxybutyrate) (P3HB), containing 3~5 carbon atoms per monomer and displaying high crystallinity and stiffness, and medium-chain-length PHAs (mcl-PHAs), often incorporating longer side-chain monomers or comonomers like 4-hydroxybutyrate (4HB), exhibiting low crystallinity, enhanced elasticity, and improved processability [128].

The widespread interest in PHAs arises from their exceptional combination of properties that meet the functional demands of both industrial and biomedical sectors. Foremost among these is their complete biodegradability in a wide range of environments. PHAs can undergo microbial degradation in soil, freshwater, and seawater, making them suitable for applications prone to environmental leakage, such as agricultural films or single-use marine gear [129,130,131]. Their biodegradation results in CO_2_, water, and biomass under aerobic conditions, and methane under anaerobic conditions, without leaving toxic residues in the environment [129,130]. This property distinguishes them from many other bioplastics that require specific industrial composting conditions. Furthermore, PHAs exhibit high biocompatibility, which makes them suitable for direct application in medical devices, tissue engineering, and controlled DDS.

In addition to their biological advantages, PHAs possess favorable thermal and mechanical properties. For example, scl-PHAs, such as P3HB, demonstrate thermoplastic behavior and can be processed using standard melt-processing techniques, offering tensile strength values in the range of 20~40 MPa and Young’s modulus up to 3.5 GPa [132]. Their crystallinity can be leveraged to produce rigid and shape-retaining structures. On the other hand, mcl-PHAs offer improved flexibility and impact resistance due to their low crystallinity, thereby extending their utility in applications requiring mechanical ductility [123,126]. Another notable feature of PHAs lies in their synthetic tunability. By adjusting the type and ratio of monomer units through microbial or enzymatic control, their degradation rate, thermal transition behavior, and mechanical response can be tailored to suit specific end-use requirements [133,134]. In addition, their relatively good gas barrier properties have made them suitable for food packaging applications where preservation of shelf life is critical [135,136]. These advantages, taken together, position PHAs as promising candidates for replacing conventional petroleum-based plastics in a wide spectrum of sustainable material applications.

#### 2.6.2. Limitations and Recent Advances

Despite these beneficial attributes, PHAs exhibit several limitations that restrict their broader industrial application. These limitations include low thermal stability, significant brittleness, slow crystallization rates, and high production costs associated with substrate and processing requirements. For example, P3HB, possessing a high degree of crystallinity (50~90%), generally displays low impact resistance and an elongation at break typically below 15%. Moreover, its glass transition temperature is reported to be approximately 4~7 °C, and the melting temperature lies between 175~180 °C, which restricts its processability using conventional thermal methods [137].

To overcome the inherent brittleness of P3HB, copolymerization with other hydroxyalkanoates has been employed as a viable strategy to enhance ductility while preserving biodegradability. For instance, PHBV films with increasing 3HV contents were systematically investigated [138]. As 3HV content increased from 3 mol% to 28 mol%, the elongation at break markedly improved from 7% to 120%, while tensile modulus decreased from 1383 MPa to 371 MPa, reflecting enhanced flexibility due to reduced crystallinity and increased amorphous segments. This enhancement in ductility makes PHBV a more suitable candidate for applications such as packaging and flexible films [138]. Beyond 3HV incorporation, additional monomers such as 4HB, 3-hydroxyhexanoate (3HHx), and 3-hydroxyoctanoate (3HO) have been explored to further tailor the mechanical and thermal properties of PHAs [132]. Incorporation of 3HHx, for instance, can drastically reduce the crystallinity of the resulting copolymer. At a composition of 12.5 mol% 3HHxE, the resulting material exhibited an amorphous nature and a remarkable elongation at break exceeding 1100%, indicating exceptional flexibility [139]. Similarly, the inclusion of mcl-monomers like 3HHx and 3HO decreases stiffness and melting temperature while enhancing impact resistance and processability [133]. These structural modifications extend the property window of PHAs from rigid, brittle materials to soft, elastic thermoplastics, enabling broader application potential in packaging, films, biomedical devices, and even elastomeric products.

In addition to mechanical and processing limitations, PHAs also face several technological and economic challenges that hinder their widespread adoption. One of the primary obstacles is their high production cost, which stems from several factors, such as the expense of microbial fermentation, the price and availability of suitable carbon sources, typically low polymer production yields from fermentation processes, and the complexity of downstream processing required to extract and purify the polymer from microbial cells. These challenges collectively make PHAs significantly more expensive to produce compared to conventional plastics, with production costs estimated to be 3~5 times higher than those of petroleum-based polymers.

Metabolic engineering and fermentation optimization have been actively pursued to address these challenges. Recent studies have demonstrated the use of non-sterile fermentation using halophilic bacteria, which reduces operational costs and contamination risks [126]. Furthermore, the integration of waste-derived feedstocks such as crude glycerol, molasses, or food processing residues has shown promise in lowering substrate costs. For example, optimized fed-batch fermentation with waste-derived carbon sources has achieved remarkably high PHA accumulation levels [140]. In one case, biopolymer content reached up to 38.9% of the cell dry weight, with a productivity of 0.34 g·L^−1^·h^−1^ using crude glycerol [141]. Likewise, a cyclic fed-batch strategy employing glucose-rich hydrolysate enabled intracellular PHA accumulation of 68.0%, resulting in a polymer concentration of 14.28 g·L^−1^ [140]. Further progress has been made in the area of recovery and purification. Environmentally benign extraction techniques using supercritical fluids, enzymatic digestion, or aqueous surfactants are being developed to replace traditional chloroform-based methods [133]. These strategies not only reduce environmental impact but also enhance the purity and molecular integrity of the recovered polymer.

Another critical challenge that remains is the ability to control the molecular weight and PDI of PHAs. Unlike synthetic polymers, where polymerization conditions can be finely tuned to achieve narrow PDIs and consistent chain lengths, PHAs produced by biological systems often exhibit batch-to-batch variability. This inconsistency affects mechanical performance, processing behavior, and application-specific properties. Efforts to standardize fermentation conditions, develop genetically stable production strains, and monitor intracellular enzymatic activities are ongoing to mitigate this variability.

Perspectively, the development of next-generation PHAs will likely focus on copolymer design, blending with complementary polymers, and the formation of nanocomposites. For instance, PHA/PLA blends can combine the toughness of PHA with the high modulus and transparency of PLA, while PHA/nanocellulose composites offer improved mechanical and barrier performance [136]. Blending strategies must address compatibility issues through reactive compatibilization or surface modification of fillers. To further unlock the potential of PHAs, future research should prioritize: (i) cost reduction via use of non-food, lignocellulosic feedstocks; (ii) strain engineering to improve yields under non-sterile and continuous fermentation; (iii) scalable and green recovery processes; and (iv) standardization of polymer quality in terms of molecular weight and dispersity. Through these advancements, PHAs could play a pivotal role in transitioning toward a circular and sustainable polymer economy.

**Table 2:** Mechanical, thermal, and barrier properties of representative biodegradable plastics ^a^.

Biodegradable Plastic	Tensile Strength (MPa)	Young’s Modulus (GPa)	T_g_ (°C)	T_m_ (°C)	Elongation at Break (%)	Oxygen Permeability (OP) (cm^3^∙mm/m^2^∙Day∙atm)	Water Vapor Permeability (WVP) (g∙mm/m^2^∙Day∙kpa)	Substitutes for Conventional Plastic	Ref.
PLA	21~60	0.35~3.5	45~60	150~180	2~10	132~590 (23 °C/RH 50% or 0%) [142]	63~342 (23 °C/RH 85%) [142,143]	PET, PS	[22]
PGA	~115	~7	35~40	220~230	~15	~1 (30 °C/RH 0%) [144]	~10 (40 °C/RH 90%) [144]	PET, Nylon	[18]
PCL	10.5~27.3	0.2~0.4	~−60	56~65	80~800	1990 (25 °C/RH 0%) [145]	173 (23 °C/RH 75%) [146] 137 (35 °C/RH 48%) [147]	LDPE	[66]
PBS	~40	~0.7	~−15	~115	~230	208 (23 °C/RH 50%) [148] 340 (25 °C/RH 90%) [149]	175 (25 °C) [150]	LDPE, PP	[82,86]
PBAT	~21	0.02~0.035	~−30	115~125	~700	2440 (23 °C/RH 50%) [151]	1380 (23 °C/RH 100%) [151]	LDPE/LLDPE	[107]
PHA (P3HB) ^b^	15~40	1~3.5	4~9	165~180	1~15	8 (23 °C/RH 85%) [152] 85 (23 °C/RH 0%) [153] 230 (25 °C/RH 80%) [154]	106 (23 °C/RH 50%) [152] 30 (25 °C/RH 100%) [155] 26 (37.8 °C/RH 100%) [154]	PE, PP, PS	[132,137]

^a^ The considerable variability in the numerical values presented in the table is attributed to the use of data reported in multiple independent studies, where differences in molecular weight, crystallinity, and other physicochemical properties, along with diverse experimental parameters, contribute to the observed ranges. ^b^ Properties shown are for poly(3-hydroxybutyrate) (P3HB), which serves as a representative example of the PHAs family. Note that PHAs properties vary significantly with monomer composition and molecular structure.

## 3. Degradation Behavior and Standard Evaluation Methods

**Figure 8:**
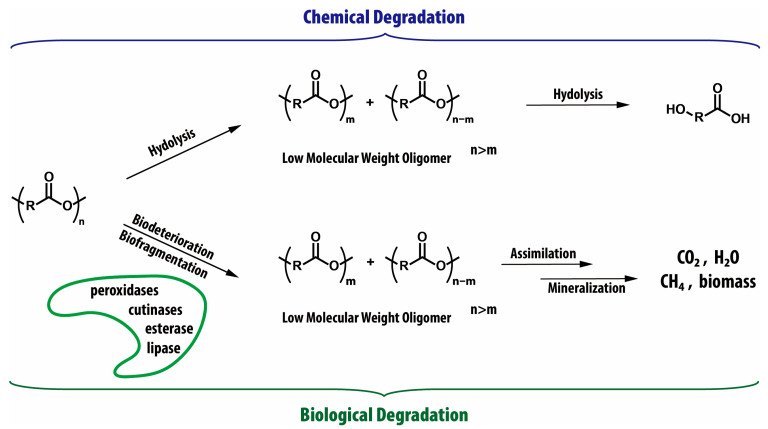
Degradation pathways of bioplastics: chemical and biological mechanisms.

The degradation of biodegradable plastics occurs through both chemical and biological pathways (Figure 8). Chemical processes, such as hydrolysis, break down polymer chains into low molecular weight compounds, while biological processes not only cleave polymers but also further metabolize these small molecules into CO_2_, H_2_O, CH_4_, and biomass [156]. A fundamental characteristic of most biodegradable plastics is their polyester backbone, which contains hydrolytically labile ester bonds [131,156]. These ester linkages serve as primary degradation sites, enabling both abiotic and biotic cleavage of the polymer chains. In the initial chemical degradation pathway, water molecules cleave ester bonds through hydrolysis, especially under elevated temperature and humidity, yielding oligomers and monomers [157]. This step can be catalyzed under acidic and basic conditions, which further accelerate hydrolytic cleavage [158]. On the other hand, biological degradation begins as microorganisms excrete extracellular enzymes, such as esterases, lipases, cutinases, and peroxidases, that catalyze the cleavage of polymer chains into smaller fragments, which are subsequently assimilated and metabolized into CO_2_ and H_2_O under aerobic conditions, or CH_4_ under anaerobic conditions [159,160]. The rate and extent of biodegradation are highly dependent on environmental parameters such as temperature, humidity, pH, and microbial diversity [159]. In addition to external conditions, polymer morphology, especially crystallinity and chain packing, significantly influences the degradability. Polymers with tightly packed, highly ordered crystalline domains, such as PLA and polyethylene terephthalate (PET), hinder water diffusion and enzymatic penetration, thereby preventing degradation. Crystalline regions act as physical barriers to hydrolysis and microbial attack, while amorphous regions are more susceptible [161]. For example, although PLA contains a hydrolyzable ester backbone, its high glass transition temperature and semi-crystalline structure limit degradation under ambient conditions, requiring elevated temperatures and controlled environments for effective breakdown [162].

Environmental factors become especially important when considering the biodegradation of bioplastics in different disposal environments such as landfills, composting facilities, and natural ecosystems (Table 3). In anaerobic landfill conditions, degradation is notably hindered due to the absence of oxygen and limited microbial activity. For instance, studies have shown negligible mass loss of PLA even after one year under landfill-like conditions [159]. Furthermore, anaerobic decomposition leads to CH_4_ production, which has a global warming potential 28 to 83 times greater than that of CO_2_, depending on the time horizon considered, and this significantly raises environmental concerns [163]. This emphasizes the importance of proper landfill management and gas capture systems to minimize emissions. Standardized methods such as ASTM D5511 and ISO 15985 are employed to assess the biodegradability of plastics under anaerobic conditions that simulate landfill environments [164,165]. ASTM D5511 evaluates the extent and rate of anaerobic biodegradation of plastic materials in accelerated landfill conditions by measuring biogas (primarily CH_4_ and CO_2_) production over time. Similarly, ISO 15985 provides a framework for determining the ultimate anaerobic biodegradability of plastics under high-solids anaerobic digestion conditions, using controlled laboratory-scale reactors [9]. These methods are essential for understanding the environmental fate of bioplastics in landfill scenarios, where oxygen is limited.

**Table 3:** International standards and test methods for evaluating biodegradation of bioplastic materials.

Environment	Standard/ Test Method	Analysis Time (Months)	Parameters Monitored	Interpretation of Results	Validity Criteria
Soil	ASTM D5988 [166]	Up to 6	CO_2_ evolution, weight loss, visual appearance	- Aerobic biodegradation in soil; high CO_2_ evolution and weight loss suggest biodegradability.	- Control must degrade; no microbial inhibition by the test sample.
	ISO 17556 [167]	Up to 24	CO_2_ evolution, weight loss, visual disintegration	- Aerobic biodegradation in soil compared to positive control.	- Positive control must show clear degradation.
	ISO 23517 [168]	Variable	CO_2_ evolution, ecotoxicity	- Aerobic biodegradation and toxicity evaluation.	- No toxic effect; significant biodegradation required.
Composting	ASTM D5338 [169]	Up to 6	CO_2_ evolution, weight loss, disintegration	- Degree of aerobic biodegradation under controlled composting conditions. - High CO_2_ evolution and disintegration indicate compostability.	- Positive control shows significant biodegradation. - Test material should not inhibit microbial activity. - Disintegration requirements apply.
	ISO 14855 [170]	Up to 6			- Positive control shows significant biodegradation. - Toxicity tests should be passed. - Disintegration criteria (e.g., <10% remains on a 2 mm sieve).
	ASTM D6400 [171]	Up to 6	CO_2_ evolution, weight loss, disintegration, plant toxicity	- Specification for labeling plastics designed to be aerobically composted. - High CO_2_ evolution and disintegration indicate compostability.	- Material must achieve 90% biodegradation within 180 days. - Disintegration and plant toxicity criteria must be met.
	ISO 17088 [172]	Up to 6		- Specification for compostable plastics under aerobic conditions. - High CO_2_ evolution and disintegration indicate compostability.	
Aquatic (freshwater)	ISO 14851 [173]	Up to 6	CO_2_ evolution, dissolved organic carbon (DOC)	- Degree of aerobic biodegradation in an aqueous environment. - Higher CO_2_ evolution and decrease in DOC indicate higher biodegradability.	- Positive control shows significant biodegradation. - Test material should not be toxic to aquatic microorganisms.
Aquatic (marine)	ASTM D6691 [174]	Up to 6	CO_2_ evolution, dissolved organic carbon (DOC)	- Degree of aerobic biodegradation in a marine environment. - Higher CO_2_ evolution and decrease in DOC indicate higher biodegradability.	- Positive control shows significant biodegradation using a defined microbial consortium. - Test material should not be toxic to marine microorganisms.
	ISO 19679 [175]	Up to 6		- Degree of aerobic biodegradation in marine sediment. - Higher CO_2_ evolution and decrease in DOC indicate higher biodegradability.	- Positive control shows significant biodegradation. - Test material should not be toxic to marine microorganisms.
	ISO 18830 [176]	Up to 6		- Degree of aerobic biodegradation in marine environments. - Higher CO_2_ evolution and decrease in DOC indicate higher biodegradability.	
Anaerobic digestion	ASTM D5511 [164]	Up to 2	Biogas production (CH_4_, CO_2_), weight loss	- Degree of anaerobic biodegradation under highsolids anaerobicdigestion conditions. - Higher biogas production and weight loss indicate higher biodegradability.	- Positive control shows significant biodegradation. - Test material should not inhibit anaerobic microbial activity.
	ISO 15985 [165]	15 days (extendable until plateau is reached)	Biogas (CH_4_, CO_2_) evolution, volatile solids reduction, vis-ual disintegration	- Degree of anaerobic biodegradation under accelerated landfill conditions. - Higher biogas production and weight loss indicate higher biodegradability.	- Positive control (e.g., cellulose) must show clear degradation; no inhibition of anaerobic microbial activity.

In contrast, industrial composting provides a more effective route for bioplastic degradation. Among the biodegradable plastics discussed in this review, PGA, PCL, PBAT, and PHA can exhibit substantial decomposition within days to weeks under controlled composting conditions, where elevated temperature, high humidity, and robust microbial populations drive rapid breakdown. However, PLA and PBS degrade more slowly, requiring higher temperatures and humidity for effective breakdown. It is worth noting that the physical form of plastics, such as films, powders, or granules, can substantially influence their degradability, as it affects surface area, porosity, and microbial accessibility. To verify composting performance, a suite of international standards is employed to evaluate different aspects of biodegradation, disintegration, and overall compostability. ASTM D5338 and ISO 14855 are laboratory-scale methods that assess the aerobic biodegradation of plastics under controlled composting conditions by measuring the evolution of CO_2_ [169,170]. ISO 16929 focuses on the physical disintegration of plastics in simulated composting environments, determining whether the material breaks down into fragments small enough not to be visible or manually separable after composting [9,177]. ASTM D6400 and ISO 17088 provide comprehensive criteria for certifying plastic materials as compostable, encompassing not only biodegradation and disintegration but also chemical safety and the absence of ecotoxic effects [9,171,172]. Together, these standards ensure that bioplastics meet the necessary performance requirements for industrial composting systems, supporting their safe integration into waste management streams.

Marine environments, on the other hand, present unique challenges. The degradation of bioplastics such as PLA is significantly slower in seawater due to lower temperatures, lower microbial activity, and the dilution effect of the aquatic medium. Partial decomposition may result in microplastic formation, posing ecological risks. Although certain marine microbes capable of attacking bioplastics have been identified, their activity is far less efficient than in terrestrial environments. This highlights the need for new bioplastics or blends designed for enhanced degradation in aquatic ecosystems. Assessment protocols such as ISO 18830, ISO 19679, and ASTM D6691 are employed to evaluate the biodegradability of plastics in marine environments, each targeting different aspects of degradation under seawater conditions [174,175,176]. ISO 18830 measures aerobic biodegradation based on CO_2_ evolution in seawater, providing insight into microbial mineralization over time. ISO 19679 assesses the degree of biodegradation of plastics by monitoring oxygen demand in closed respirometric systems under marine sediment interface conditions, offering a simulation of more benthic environments. ASTM D6691 evaluates aerobic biodegradation of plastic materials in seawater using a similar respirometric approach. These standardized tests are essential for understanding how bioplastics behave in aquatic ecosystems and play a critical role in guiding the design and development of materials that minimize long-term persistence and ecological harm in marine environments.

In summary, the efficiency of bioplastic degradation is context-dependent, governed by both intrinsic polymer properties, such as crystallinity, hydrophobicity, and molecular weight, and extrinsic environmental factors, including microbial diversity, temperature, moisture, and oxygen availability. Standardized test methods play an important role in providing reliable and comparable data on biodegradability across disposal scenarios. Continued research and development are essential to enhance the environmental compatibility of bioplastics and to tailor their degradation profiles to the ecosystems they are most likely to encounter at EOL.

## 4. Conclusions and Perspectives

Plastics have become indispensable to modern society due to their unmatched versatility, durability, and cost-effectiveness. However, their widespread use has led to persistent environmental problems, including plastic pollution and greenhouse gas emissions, necessitating urgent intervention through sustainable alternatives. Bioplastics, particularly PLA, PBS, PBAT, and PHAs, have emerged as promising candidates owing to their biodegradability, bio-based origin, and compatibility with existing processing techniques.

Nonetheless, several structural and systemic barriers continue to limit the large-scale adoption of bioplastics. In terms of material properties, many biopolymers struggle to achieve the mechanical strength, thermal stability, and application diversity of conventional plastics, especially in demanding sectors such as automotive, electronics, and high-performance applications. The degradation behavior of bioplastics is also highly environment-dependent, often requiring industrial composting conditions to attain complete mineralization. These material limitations are further compounded by economic challenges, including the high cost of bio-based feedstocks, complex fermentation processes, and energy- or solvent-intensive downstream recovery. These factors result in production costs that remain several times higher than those of petroleum-derived plastics, limiting market competitiveness.

To enhance performance and overcome functional limitations, recent research has actively explored polymer design strategies, including copolymerization, blending, compatibilization, and nanocomposite engineering. These approaches have led to notable improvements in mechanical ductility, barrier performance, and degradation kinetics. Simultaneously, biotechnological and chemical innovations are reshaping approaches to monomer sourcing. The production of monomers, such as SA, AA, BDO, and other traditionally fossil-derived monomers, from renewable biomass sources offers a sustainable alternative that supports a pathway toward carbon neutrality while enhancing supply chain resilience through dual-sourcing flexibility. Recent advances in enzyme engineering also offer novel recycling possibilities. For instance, the recent development of a thermostable PETase (Kubu-P^M12^), which achieved over 90% depolymerization of post-consumer PET at high solid loadings within eight hours at elevated temperatures, demonstrates exceptional potential for real-world biocatalytic PET recycling [178]. In addition to bio-recycling, embedding enzymes directly into biodegradable polymers has recently emerged as a transformative strategy [179]. A masterbatch-based melt-extrusion process has enabled enzyme-embedded plastics, including PLA, PCL, PBS, PBAT, and PHAs, to undergo rapid self-degradation under aqueous and composting conditions. Notably, an engineered PLA hydrolase incorporated at only 0.02 wt% into PLA facilitated complete degradation under home-composting conditions within 20~24 weeks [180]. These results demonstrate that enzyme-embedded systems provide a novel strategy to achieve controlled and on-demand biodegradability in bioplastics.

However, advancements in material performance and catalytic technologies alone are insufficient to fully realize the potential of bioplastics. Without standardized waste collection and composting infrastructure tailored to the EOL pathways of bioplastics, their environmental benefits may be compromised. The incompatibility of many bioplastics with current mechanical recycling systems presents a critical barrier, often leading to contamination or downcycling. Therefore, a comprehensive system-level shift across material innovation, waste management, and regulation is essential. Regulatory frameworks must support bioplastics through clear definitions, certifications, and market incentives. Industries need to collaborate on improving design-for-recycling, while consumers must be engaged in proper usage and disposal behaviors.

In this context, bioplastics should not be viewed as a standalone solution but as an integral part of a multifaceted strategy to transition toward a circular plastics economy. The future of sustainable plastics lies at the intersection of materials innovation, bioprocessing, catalytic recycling, and systemic policy support. Ongoing research should prioritize scalable and cost-efficient synthetic routes, robust life-cycle assessments (LCA), and the development of closed-loop recovery systems that preserve material value. If these challenges are collectively addressed, bioplastics hold strong potential to reduce the environmental footprint of polymers and significantly contribute to global sustainability goals.

## Figures and Tables

**Table 1:** Comparison of polycondensation and ring-opening polymerization for polyester synthesis [24].

	Polycondensation	Ring-Opening Polymerization
Monomer	Diacids + diols/hydroxyacids	Cyclic monomers
Cost	Cheap, Readily available	Expensive (need cyclic monomers)
Growth pathway	Step growth	Chain growth
Driving force	Removal of condensates	Release of ring strain
Process	High temp., Reduced pressure	Lower temp.
Molecular Weight	Low, Limited	High, Controllable
Molecular Weight Distribution	Broad	Narrow

## Data Availability

No new data were created or analyzed in this study. Data sharing is not applicable to this article.

## References

[1] Nanda S., Patra B.R., Patel R., Bakos J., Dalai A.K. (2022). Innovations in Applications and Prospects of Bioplastics and Biopolymers: A Review. Environ. Chem. Lett..

[2] Statista (2024). Global Plastic Production 1950–2023.

[3] Geyer R., Jambeck J.R., Law K.L. (2017). Production, Use, and Fate of All Plastics Ever Made. Sci. Adv..

[4] Wang J., Tan Z., Peng J., Qiu Q., Li M. (2016). The Behaviors of Microplastics in the Marine Environment. Mar. Environ. Res..

[5] Jung U., Chae E., Choi S.-S. (2024). Characteristics of Tire-Road Wear Particles Produced on Indoor Parking Garage Ramp. Elastom. Compos..

[6] da Costa J.P., Santos P.S.M., Duarte A.C., Rocha-Santos T. (2016). (Nano)plastics in the Environment—Sources, Fates and Effects. Sci. Total Environ..

[7] Iwata T. (2015). Biodegradable and Bio-Based Polymers: Future Prospects of Eco-Friendly Plastics. Angew. Chem. Int. Ed..

[8] Sinha Ray S., Bousmina M. (2005). Biodegradable Polymers and Their Layered Silicate Nanocomposites: In Greening the 21st Century Materials World. Prog. Mater. Sci..

[9] Silva R.R.A., Marques C.S., Arruda T.R., Teixeira S.C., de Oliveira T.V. (2023). Biodegradation of Polymers: Stages, Measurement, Standards and Prospects. Macromol.

[10] De Falco F., Avolio R., Errico M.E., Di Pace E., Avella M., Cocca M., Gentile G. (2021). Comparison of Biodegradable Polyesters Degradation Behavior in Sand. J. Hazard. Mater..

[11] Karan H., Funk C., Grabert M., Oey M., Hankamer B. (2019). Green Bioplastics as Part of a Circular Bioeconomy. Trends Plant Sci..

[12] Braskem S.A. (2024). Integrated Report 2024.

[13] Cucina M., de Nisi P., Tambone F., Adani F. (2021). The Role of Waste Management in Reducing Bioplastics’ Leakage into the Environment: A Review. Bioresour. Technol..

[14] Titone V., Botta L., La Mantia F.P. (2024). Mechanical Recycling of New and Challenging Polymer Systems: A Brief Overview. Macromol. Mater. Eng..

[15] Fredi G., Dorigato A. (2021). Recycling of Bioplastic Waste: A Review. Adv. Ind. Eng. Polym. Res..

[16] Muller J., González-Martínez C., Chiralt A. (2017). Combination of Poly(lactic) Acid and Starch for Biodegradable Food Packaging. Materials.

[17] European Bioplastics e.V. (2024). Bioplastics Market Development Update 2024.

[18] Jem K.J., Tan B. (2020). The Development and Challenges of Poly (lactic acid) and Poly (glycolic acid). Adv. Ind. Eng. Polym. Res..

[19] Li X., Lin Y., Liu M., Meng L., Li C. (2023). A Review of Research and Application of Polylactic acid Composites. J. Appl. Polym. Sci..

[20] Singhvi M., Gokhale D. (2013). Biomass to Biodegradable Polymer (PLA). RSC Adv..

[21] Abedi E., Hashemi S.M.B. (2020). Lactic acid Production—Producing Microorganisms and Substrates Sources-State of Art. Heliyon.

[22] Taib N.-A.A.B., Rahman M.R., Huda D., Kuok K.K., Hamdan S., Bakri M.K.B., Julaihi M.R.M.B., Khan A. (2023). A Review on Poly-lactic acid (PLA) as a Biodegradable Polymer. Polym. Bull..

[23] Tsuji H., Ikada Y. (1996). Crystallization from the Melt of Poly(lactide)s with Different Optical Purities and Their Blends. Macromol. Chem. Phys..

[24] Pang K., Kotek R., Tonelli A. (2006). Review of Conventional and Novel Polymerization Processes for Polyesters. Prog. Polym. Sci..

[25] Lasprilla A.J.R., Martinez G.A.R., Lunelli B.H., Jardini A.L., Filho R.M. (2012). Poly-lactic acid Synthesis for Application in Biomedical devices—A Review. Biotechnol. Adv..

[26] Trivedi A.K., Gupta M.K., Singh H. (2023). PLA Based Biocomposites for Sustainable Products: A Review. Adv. Ind. Eng. Polym. Res..

[27] Nizamuddin S., Chen C. (2024). Biobased, Biodegradable and Compostable Plastics: Chemical Nature, Biodegradation Pathways and Environmental Strategy. Environ. Sci. Pollut. Res..

[28] Gigante V., Canesi I., Cinelli P., Coltelli M.B., Lazzeri A. (2019). Rubber Toughening of Polylactic Acid (PLA) with Poly(butylene adipate-co-terephthalate) (PBAT): Mechanical Properties, Fracture Mechanics and Analysis of Ductile-to-Brittle Behavior while Varying Temperature and Test Speed. Eur. Polym. J..

[29] Harada M., Iida K., Okamoto K., Hayashi H., Hirano K. (2008). Reactive Compatibilization of Biodegradable Poly(lactic acid)/Poly(ε-caprolactone) Blends with Reactive Processing Agents. Polym. Eng. Sci..

[30] Van de Perre D., Serbruyns L., Coltelli M.-B., Gigante V., Aliotta L., Lazzeri A., Geerinck R., Verstichel S. (2024). Tuning Biodegradation of Poly (lactic acid) (PLA) at Mild Temperature by Blending with Poly (butylene succinate-co-adipate) (PBSA) or Polycaprolactone (PCL). Materials.

[31] Ojijo V., Ray S.S. (2015). Super Toughened Biodegradable Polylactide Blends with Non-linear Copolymer Interfacial Architecture Obtained via Facile In-situ Reactive Compatibilization. Polymer.

[32] Nofar M., Heuzey M.C., Carreau P.J., Kamal M.R. (2016). Effects of Nanoclay and Its Localization on the Morphology Stabilization of PLA/PBAT Blends under Shear Flow. Polymer.

[33] Li Y., Huang L., Zhou S., Li J., Qi C., Tao H. (2024). Synthesis and Investigation of Sustainable Long-chain Branched Poly(lactic acid-r-malic acid) Copolymer as Toughening Agent for PLA Blends. Polymer.

[34] Choi J.-Y., Lee Y.-J., Park S., Kim C.-W., Kang D.-G. (2021). Transparent Poly(Lactic Acid-b-3-Hydroxypropionic Acid) Block Copolymer and Articles Including the Same.

[35] Imai Y., Tominaga Y., Tanaka S., Yoshida M., Furutate S., Sato S., Koh S., Taguchi S. (2024). Modification of Poly(lactate) via Polymer Blending with Microbially Produced Poly[(R)-lactate-co-(R)-3-hydroxybutyrate] Copolymers. Int. J. Biol. Macromol..

[36] Oh J.K. (2011). Polylactide (PLA)-based Amphiphilic Block Copolymers: Synthesis, Self-assembly, and Biomedical Applications. Soft Matter.

[37] Zhao X., Liu J., Li J., Liang X., Zhou W., Peng S. (2022). Strategies and Techniques for Improving Heat Resistance and Mechanical Performances of Poly(lactic acid) (PLA) Biodegradable Materials. Int. J. Biol. Macromol..

[38] Zhang T., Wang X., Jiang J., Srithep Y., Li Q. (2025). Enhancing Heat Resistance of PBAT Foams by Incorporating sc-PLA and In Situ Fibrillation Process. ACS Appl. Polym. Mater..

[39] Petchwattana N., Covavisaruch S., Petthai S. (2014). Influence of Talc Particle Size and Content on Crystallization Behavior, Mechanical Properties and Morphology of Poly(lactic acid). Polym. Bull..

[40] Samantaray P.K., Little A., Haddleton D.M., McNally T., Tan B., Sun Z., Huang W., Ji Y., Wan C. (2020). Poly(glycolic acid) (PGA): A Versatile Building Block Expanding High Performance and Sustainable Bioplastic Applications. Green Chem..

[41] Sanko V., Sahin I., Aydemir Sezer U., Sezer S. (2019). A Versatile Method for the Synthesis of Poly(glycolic acid): High Solubility and Tunable Molecular Weights. Polym. J..

[42] Budak K., Sogut O., Aydemir Sezer U. (2020). A Review on Synthesis and Biomedical Applications of Polyglycolic acid. J. Polym. Res..

[43] Market Research Future (2024). Polyglycolic Acid Market Research Report—Global Forecast 2024–2032.

[44] Singh V., Tiwari M. (2010). Structure-Processing-Property Relationship of Poly(Glycolic Acid) for Drug Delivery Systems 1: Synthesis and Catalysis. Int. J. Polym. Sci..

[45] Nieuwenhuis J. (1992). Synthesis of Polylactides, Polyglycolides and Their Copolymers. Clin. Mater..

[46] Takahashi K., Taniguchi I., Miyamoto M., Kimura Y. (2000). Melt/Solid Polycondensation of Glycolic acid to Obtain High-molecular-weight Poly(glycolic acid). Polymer.

[47] Altay E., Jang Y.-J., Kua X.Q., Hillmyer M.A. (2021). Synthesis, Microstructure, and Properties of High-Molar-Mass Polyglycolide Copolymers with Isolated Methyl Defects. Biomacromolecules.

[48] Eberhart R.C., Shih-Horng S., Truong N.K., Meital Z., Liping T., Nelson K.D., Frenkel P. (2003). Review: Bioresorbable Polymeric Stents: Current Status and Future Promise. J. Biomater. Sci.-Polym. Ed..

[49] Chen S., Meng X., Xin Z., Gong W., Li C., Wen W. (2024). Preparation of Rigidity Toughness Balance and Stable Poly(glycolic acid) Based on Chain Extension Reaction. J. Appl. Polym. Sci..

[50] Ellingford C., Samantaray P.K., Farris S., McNally T., Tan B., Sun Z., Huang W., Ji Y., Wan C. (2022). Reactive Extrusion of Biodegradable PGA/PBAT Blends to Enhance Flexibility and Gas Barrier Properties. J. Appl. Polym. Sci..

[51] Ayyoob M., Lee D.H., Kim J.H., Nam S.W., Kim Y.J. (2017). Synthesis of Poly(glycolic acids) via Solution Polycondensation and Investigation of Their Thermal Degradation Behaviors. Fibers Polym..

[52] Liu Y., Bai X., Liang A. (2016). Synthesis, Properties, and In Vitro Hydrolytic Degradation of Poly(d,l-lactide-co-glycolide-co-ε-caprolactone). Int. J. Polym. Sci..

[53] Lee S.-H., Kim B.-S., Kim S.H., Choi S.W., Jeong S.I., Kwon I.K., Kang S.W., Nikolovski J., Mooney D.J., Han Y.-K. (2003). Elastic Biodegradable Poly(glycolide-co-caprolactone) Scaffold for Tissue Engineering. J. Biomed. Mater. Res. Part A.

[54] Díaz-Celorio E., Franco L., Rodríguez-Galán A., Puiggalí J. (2013). Study on the Hydrolytic Degradation of Glycolide/Trimethylene carbonate Copolymers Having Different Microstructure and Composition. Polym. Degrad. Stabil..

[55] Wang Y., Jia Z., Jiang J., Mao X., Pan X., Wu J. (2019). Highly Regioselective Ring-Opening Polymerization of Cyclic Diester for Alternating Sequence-Controlled Copolymer Synthesis of Mandelic Acid and Glycolic Acid. Macromolecules.

[56] Shen J., Wang K., Ma Z., Xu N., Pang S., Pan L. (2021). Biodegradable Blends of Poly(butylene adipate-co-terephthalate) and Polyglycolic acid with Enhanced Mechanical, Rheological and Barrier Performances. J. Appl. Polym. Sci..

[57] Xu Z., Dong Y., Yang Y., Zhu J. (2023). Mechanical, Barrier, and Biodegradable Properties of Poly(butylene adipate-co-terephthalate)/Polyglycolic Acid Blends Prepared by Reactive Extrusion. ACS Appl. Eng. Mater..

[58] Miñano J., Puiggalí J., Franco L. (2020). Effect of Curcumin on Thermal Degradation of Poly(glycolic acid) and Poly(ε-caprolactone) Blends. Thermochim. Acta.

[59] Samadi K., Francisco M., Hegde S., Diaz C.A., Trabold T.A., Dell E.M., Lewis C.L. (2019). Mechanical, Rheological and Anaerobic Biodegradation Behavior of a Poly(lactic acid) Blend Containing a Poly(lactic acid)-co-poly(glycolic acid) Copolymer. Polym. Degrad. Stabil..

[60] Ma Z., Yin T., Jiang Z., Weng Y., Zhang C. (2024). Bio-based Epoxidized Soybean Oil Branched Cardanol Ethers as Compatibilizers of Polybutylene succinate (PBS)/Polyglycolic acid (PGA) Blends. Int. J. Biol. Macromol..

[61] Wu H., Duan M., Ning Z., Gan H., Jiang N. (2025). Reactive Toughening of Poly(Glycolic Acid)/Poly(ε-Caprolactone) Blends Using Environmentally Friendly and Cost-Effective Bio-Based Chain Extenders. J. Appl. Polym. Sci..

[62] Abdel-Motaal F.F., El-Sayed M.A., El-Zayat S.A., Ito S.I. (2014). Biodegradation of Poly (ε-caprolactone) (PCL) Film and Foam Plastic by Pseudozyma japonica sp. nov., a Novel Cutinolytic Ustilaginomycetous Yeast Species. 3 Biotech.

[63] Labet M., Thielemans W. (2009). Synthesis of Polycaprolactone: A Review. Chem. Soc. Rev..

[64] Wu C., Zhang Z., Chen C., He F., Zhuo R. (2013). Synthesis of Poly(ε-caprolactone) by an Immobilized Lipase Coated with Ionic Liquids in a Solvent-free Condition. Biotechnol. Lett..

[65] Dwivedi R., Kumar S., Pandey R., Mahajan A., Nandana D., Katti D.S., Mehrotra D. (2020). Polycaprolactone as Biomaterial for Bone Scaffolds: Review of Literature. J. Oral Biol. Craniofac. Res..

[66] Kayan G.Ö., Kayan A. (2023). Polycaprolactone Composites/Blends and Their Applications Especially in Water Treatment. ChemEngineering.

[67] Bartnikowski M., Dargaville T.R., Ivanovski S., Hutmacher D.W. (2019). Degradation Mechanisms of Polycaprolactone in the Context of Chemistry, Geometry and Environment. Prog. Polym. Sci..

[68] Sung H.-J., Meredith C., Johnson C., Galis Z.S. (2004). The Effect of Scaffold Degradation Rate on Three-dimensional Cell Growth and Angiogenesis. Biomaterials.

[69] Jakus A.E., Rutz A.L., Shah R.N. (2016). Advancing the Field of 3D Biomaterial Printing. Biomed. Mater..

[70] Backes E.H., Harb S.V., Beatrice C.A.G., Shimomura K.M.B., Passador F.R., Costa L.C., Pessan L.A. (2022). Polycaprolactone Usage in Additive Manufacturing Strategies for Tissue Engineering Applications: A Review. J. Biomed. Mater. Res. Part B.

[71] Bhadran A., Shah T., Babanyinah G.K., Polara H., Taslimy S., Biewer M.C., Stefan M.C. (2023). Recent Advances in Polycaprolactones for Anticancer Drug Delivery. Pharmaceutics.

[72] Finotti P.F.M., Costa L.C., Capote T.S.O., Scarel-Caminaga R.M., Chinelatto M.A. (2017). Immiscible Poly(lactic acid)/Poly(ε-caprolactone) for Temporary Implants: Compatibility and Cytotoxicity. J. Mech. Behav. Biomed. Mater..

[73] Wachirahuttapong S., Thongpin C., Sombatsompop N. (2016). Effect of PCL and Compatibility Contents on the Morphology, Crystallization and Mechanical Properties of PLA/PCL Blends. Energy Procedia.

[74] Zhou Z., Wang E., Liang Y., Miao Y., Chen H., Ling M., Li W., Huang J., Zhang W. (2024). Bio-based PA-grafted Bamboo Charcoal for Improving the Flame Retardancy of PLA/PCL Film without Damaging Mechanical Properties and Degradability. Ind. Crop. Prod..

[75] Li Y., Han C., Yu Y., Xiao L. (2020). Effect of Loadings of Nanocellulose on the Significantly Improved Crystallization and Mechanical Properties of Biodegradable Poly(ε-caprolactone). Int. J. Biol. Macromol..

[76] Dethe M.R., Prabakaran A., Ahmed H., Agrawal M., Roy U., Alexander A. (2022). PCL-PEG Copolymer Based Injectable Thermosensitive Hydrogels. J. Control. Release.

[77] Youssouf L., Bhaw-Luximon A., Diotel N., Catan A., Giraud P., Gimié F., Koshel D., Casale S., Bénard S., Meneyrol V. (2019). Enhanced Effects of Curcumin Encapsulated in Polycaprolactone-grafted Oligocarrageenan Nanomicelles, a Novel Nanoparticle Drug Delivery System. Carbohydr. Polym..

[78] Hemmati K., Ghaemy M. (2016). Synthesis of New Thermo/pH Sensitive Drug Delivery Systems Based on Tragacanth Gum Polysaccharide. Int. J. Biol. Macromol..

[79] Gao X., Wang B., Wei X., Rao W., Ai F., Zhao F., Men K., Yang B., Liu X., Huang M. (2013). Preparation, Characterization and Application of Star-shaped PCL/PEG Micelles for the Delivery of Doxorubicin in the Treatment of Colon Cancer. Int. J. Nanomed..

[80] Rostamizadeh K., Manafi M., Nosrati H., Kheiri Manjili H., Danafar H. (2018). Methotrexate-conjugated mPEG–PCL Copolymers: A Novel Approach for Dual Triggered Drug Delivery. New J. Chem..

[81] Senevirathne S.A., Boonsith S., Oupicky D., Biewer M.C., Stefan M.C. (2015). Synthesis and Characterization of Valproic acid ester Pro-drug Micelles via an Amphiphilic Polycaprolactone Block Copolymer Design. Polym. Chem..

[82] Xu J., Guo B.-H. (2010). Poly(butylene succinate) and Its Copolymers: Research, Development and Industrialization. Biotechnol. J..

[83] Savitha K.S., Ravji Paghadar B., Senthil Kumar M., Jagadish R.L. (2022). Polybutylene succinate, a Potential Bio-degradable Polymer: Synthesis, Copolymerization and Bio-degradation. Polym. Chem..

[84] Kim J., Park S., Jung S., Yun H., Choi K., Heo G., Jin H.-J., Park S., Kwak H.W. (2023). Biodegradation Behavior of Polybutylene Succinate (PBS) Fishing Gear in Marine Sedimentary Environments for Ghost Fishing Prevention. Polym. Degrad. Stabil..

[85] Labruyère C., Talon O., Berezina N., Khousakoun E., Jérôme C. (2014). Synthesis of Poly(butylene succinate) Through Oligomerization–cyclization–ROP Route. RSC Adv..

[86] Fabbri M., Guidotti G., Soccio M., Lotti N., Govoni M., Giordano E., Gazzano M., Gamberini R., Rimini B., Munari A. (2018). Novel Biocompatible PBS-based Random Copolymers Containing PEG-like Sequences for Biomedical Applications: From Drug Delivery to Tissue Engineering. Polym. Degrad. Stabil..

[87] Nelson T.F., Baumgartner R., Jaggi M., Bernasconi S.M., Battagliarin G., Sinkel C., Künkel A., Kohler H.-P.E., McNeill K., Sander M. (2022). Biodegradation of Poly(butylene succinate) in Soil Laboratory Incubations Assessed by Stable Carbon Isotope Labelling. Nat. Commun..

[88] da Costa V.C., de Souza Pinto G.L., Nascimento M.V.F., de Campos V.E.B., de Souza Junior F.G. (2018). Poly (Butylene Succinate)-g-Poly(Hydroxypropyl Methacrylate) as a New Meloxican Delivery System. Macromol. Symp..

[89] Platnieks O., Barkane A., Ijudina N., Gaidukova G., Thakur V.K., Gaidukovs S. (2020). Sustainable Tetra Pak Recycled Cellulose / Poly(Butylene succinate) Based Woody-like Composites for a Circular economy. J. Clean Prod..

[90] Vandesteene M., Jacquel N., Saint-Loup R., Boucard N., Carrot C., Rousseau A., Fenouillot F. (2016). Synthesis of Branched Poly(butylene succinate): Structure Properties Relationship. Chin. J. Polym. Sci..

[91] Ma P., Hristova-Bogaerds D.G., Zhang Y., Lemstra P.J. (2014). Enhancement in Crystallization Kinetics of the Bacterially Synthesized Poly(β-hydroxybutyrate) by Poly(butylene succinate). Polym. Bull..

[92] Liang Z., Pan P., Zhu B., Dong T., Inoue Y. (2010). Mechanical and Thermal Properties of Poly(butylene succinate)/Plant Fiber Biodegradable Composite. J. Appl. Polym. Sci..

[93] Sheikholeslami S.N., Rafizadeh M., Taromi F.A., Shirali H., Jabbari E. (2016). Material Properties of Degradable Poly(butylene succinate-co-fumarate) Copolymer Networks Synthesized by Polycondensation of Pre-homopolyesters. Polymer.

[94] Zheng Y., Tian G., Xue J., Zhou J., Huo H., Li L. (2018). Effects of Isomorphic Poly(butylene succinate-co-butylene fumarate) on the Nucleation of Poly(butylene succinate) and the Formation of Poly(butylene succinate) Ring-banded Spherulites. CrystEngComm.

[95] Zheng L., Wang Z., Wu S., Li C., Zhang D., Xiao Y. (2013). Novel Poly(butylene fumarate) and Poly(butylene succinate) Multiblock Copolymers Bearing Reactive Carbon–Carbon Double Bonds: Synthesis, Characterization, Cocrystallization, and Properties. Ind. Eng. Chem. Res..

[96] Huang Y., Liu J., Zhang A., Zhou T. (2019). Crystallization Behavior of Poly(Tetramethylene Oxide) Influenced by the Crystallization Condition of Poly(Butylene Succinate) in Their Copolymers. J. Wuhan Univ. Technol.-Mat. Sci. Edit..

[97] Safari M., Leon Boigues L., Shi G., Maiz J., Liu G., Wang D., Mijangos C., Müller A.J. (2020). Effect of Nanoconfinement on the Isodimorphic Crystallization of Poly(butylene succinate-ran-caprolactone) Random Copolymers. Macromolecules.

[98] Zheng L., Li C., Wang Z., Wang J., Xiao Y., Zhang D., Guan G. (2012). Novel Biodegradable and Double Crystalline Multiblock Copolymers Comprising of Poly(butylene succinate) and Poly(ε-caprolactone): Synthesis, Characterization, and Properties. Ind. Eng. Chem. Res..

[99] Wang L., Zhang M., Lawson T., Kanwal A., Miao Z. (2019). Poly(butylene succinate-co-salicylic acid) Copolymers and Their Effect on Promoting Plant Growth. R. Soc. Open Sci..

[100] Hongsriphan N., Pinpueng A. (2019). Properties of Agricultural Films Prepared from Biodegradable Poly(Butylene Succinate) Adding Natural Sorbent and Fertilizer. J. Polym. Environ..

[101] Kasirajan S., Ngouajio M. (2012). Polyethylene and Biodegradable Mulches for Agricultural Applications: A Review. Agron. Sustain. Dev..

[102] Muthuraj R., Misra M., Mohanty A.K. (2018). Biodegradable Compatibilized Polymer Blends for Packaging Applications: A Literature Review. J. Appl. Polym. Sci..

[103] Zhang Y., Yu C., Chu P.K., Lv F., Zhang C., Ji J., Zhang R., Wang H. (2012). Mechanical and Thermal Properties of Basalt Fiber Reinforced Poly(butylene succinate) Composites. Mater. Chem. Phys..

[104] Díaz A., Katsarava R., Puiggalí J. (2014). Synthesis, Properties and Applications of Biodegradable Polymers Derived from Diols and Dicarboxylic Acids: From Polyesters to Poly(ester amide)s. Int. J. Mol. Sci..

[105] Jian J., Xiangbin Z., Xianbo H. (2020). An Overview on Synthesis, Properties and Applications of Poly(butylene-adipate-co-terephthalate)–PBAT. Adv. Ind. Eng. Polym. Res..

[106] Liu J., Jiang Z., Zhou Y., Wang B., Chen J., Li J., Huang L., Wang C. (2024). Hydrophilic and Biodegradable PBAT Copolyesters Prepared from Chemically Recycled Resources. ACS Appl. Polym. Mater..

[107] Burford T., Rieg W., Madbouly S. (2023). Biodegradable Poly(butylene adipate-co-terephthalate) (PBAT). Phys. Sci. Rev..

[108] Lee J., Park C., Fai Tsang Y., Andrew Lin K.Y. (2024). Towards Sustainable Production of Polybutylene Adipate Terephthalate: Non-Biological Catalytic Syntheses of Biomass-Derived Constituents. ChemSusChem.

[109] He Y., Luo Y., Yang M., Zhang Y., Zhu L., Fan M., Li Q. (2022). Selective Catalytic Synthesis of Bio-based Terephthalic Acid from Lignocellulose Biomass. Appl. Catal. A Gen..

[110] Sousa A.F., Vilela C., Fonseca A.C., Matos M., Freire C.S.R., Gruter G.-J.M., Coelho J.F.J., Silvestre A.J.D. (2015). Biobased Polyesters and Other Polymers From 2,5-Furandicarboxylic acid: A Tribute to Furan Excellency. Polym. Chem..

[111] Hu H., Lin C., Luan Q., Jiang X., Zhang X., Wang Q., Dong Y., Wei J., Wang J., Zhu J. (2023). Synergistic Modification of PBT with Diglycolic Acid and Succinic Acid: Fast Crystallization and High Strength-Toughness Copolyesters for Environmentally Degradable Packaging. ACS Sustain. Chem. Eng..

[112] Kijchavengkul T., Auras R., Rubino M., Selke S., Ngouajio M., Fernandez R.T. (2010). Biodegradation and Hydrolysis Rate of Aliphatic Aromatic Polyester. Polym. Degrad. Stabil..

[113] Denial M., Karthikeyan S., Godse R., Gupta V.K. (2021). Poly(butylene adipate-co-terephthalate) Polyester Synthesis Process and Product Development. Polym. Sci. Ser. C.

[114] Liu J., Jiang Z., Xie W., Wang B., Chen J., Song S., Li J., Wang C. (2024). Preparation of PBAT Copolyesters with Flame Retardant and Degradable Functions through PBT Chemical Alcoholysis and Closed-Loop Recycling. ACS Sustain. Chem. Eng..

[115] Wu D., Huang A., Fan J., Xu R., Liu P., Li G., Yang S. (2021). Effect of Blending Procedures and Reactive Compatibilizers on the Properties of Biodegradable Poly(butylene adipate-co-terephthalate)/Poly(lactic acid) Blends. J. Polym. Eng..

[116] Dammak M., Fourati Y., Tarrés Q., Delgado-Aguilar M., Mutjé P., Boufi S. (2020). Blends of PBAT with Plasticized Starch for Packaging Applications: Mechanical properties, Rheological Behaviour and Biodegradability. Ind. Crop. Prod..

[117] Wei C., Guo P., Lyu M., Wang B., Li C., Sang L., Wei Z. (2023). High Barrier Poly(Glycolic Acid) Modified Poly(Butylene Adipate-co-terephthalate) Blown Films and Accelerated Ultraviolet Degradability Evaluation. ACS Appl. Polym. Mater..

[118] Dai Z., Li B., He M., Li J., Zou X., Wang Y., Shan Y. (2024). Biodegradable and High-performance Composites of Poly (butylene adipate-co-terephthalate) with PHBV Chain-Functionalized Nanocellulose. Prog. Org. Coat..

[119] da Costa F.A.T., Parra D.F., Cardoso E.C.L., Güven O. (2023). PLA, PBAT, Cellulose Nanocrystals (CNCs), and Their Blends: Biodegradation, Compatibilization, and Nanoparticle Interactions. J. Polym. Environ..

[120] Debeli D.K., Wu L., Huang F. (2023). PBAT-based Biodegradable Nanocomposite Coating Films with Ultra-high Oxygen Barrier and Balanced Mechanical Properties. Polym. Degrad. Stabil..

[121] Dong Y., Wang J., Yang Y., Wang Q., Zhang X., Hu H., Zhu J. (2022). Bio-based Poly(butylene diglycolate-co-furandicarboxylate) Copolyesters with Balanced Mechanical, Barrier and Biodegradable Properties: A Prospective Substitute for PBAT. Polym. Degrad. Stabil..

[122] Reis M.A.M., Serafim L.S., Lemos P.C., Ramos A.M., Aguiar F.R., Van Loosdrecht M.C.M. (2003). Production of Polyhydroxyalkanoates by Mixed Microbial Cultures. Bioprocess Biosyst. Eng..

[123] Samrot A.V., Samanvitha S.K., Shobana N., Renitta E.R., Senthilkumar P., Kumar S.S., Abirami S., Dhiva S., Bavanilatha M., Prakash P. (2021). The Synthesis, Characterization and Applications of Polyhydroxyalkanoates (PHAs) and PHA-Based Nanoparticles. Polymers.

[124] Chien Bong C.P., Alam M.N.H.Z., Samsudin S.A., Jamaluddin J., Adrus N., Mohd Yusof A.H., Muis Z.A., Hashim H., Salleh M.M., Abdullah A.R. (2021). A Review on the Potential of Polyhydroxyalkanoates Production from Oil-based Substrates. J. Environ. Manag..

[125] Sohail R., Jamil N., Ali I., Munir S. (2020). Animal Fat and Glycerol Bioconversion to Polyhydroxyalkanoate by Produced Water Bacteria. e-Polymers.

[126] Zytner P., Kumar D., Elsayed A., Mohanty A., Ramarao B.V., Misra M. (2023). A Review on Polyhydroxyalkanoate (PHA) Production Through the Use of Lignocellulosic Biomass. RSC Sustain..

[127] Thamarai P., Vickram A.S., Saravanan A., Deivayanai V.C., Evangeline S. (2024). Recent Advancements in Biosynthesis, Industrial Production, and Environmental Applications of Polyhydroxyalkanoates (PHAs): A Review. Bioresour. Technol. Rep..

[128] Szacherska K., Moraczewski K., Rytlewski P., Czaplicki S., Ciesielski S., Oleskowicz-Popiel P., Mozejko-Ciesielska J. (2022). Polyhydroxyalkanoates Production from Short and Medium Chain Carboxylic Acids by Paracoccus Homiensis. Sci. Rep..

[129] Nygaard D., Yashchuk O., Hermida É.B. (2021). PHA Granule Formation and Degradation by Cupriavidus Necator Under Different Nutritional Conditions. J. Basic Microbiol..

[130] Zhou Y., Lin L., Wang H., Zhang Z., Zhou J., Jiao N. (2020). Development of a CRISPR/Cas9n-based Tool for Metabolic Engineering of Pseudomonas Putida for Ferulic Acid-to-polyhydroxyalkanoate Bioconversion. Commun. Biol..

[131] Jeon Y., Jin H., Kong Y., Cha H.-G., Lee B.W., Yu K., Yi B., Kim H.T., Joo J.C., Yang Y.-H. (2023). Poly(3-hydroxybutyrate) Degradation by *Bacillus infantis* sp. Isolated from Soil and Identification of phaZ and bdhA Expressing PHB Depolymerase. J. Microbiol. Biotechnol..

[132] McAdam B., Brennan Fournet M., McDonald P., Mojicevic M. (2020). Production of Polyhydroxybutyrate (PHB) and Factors Impacting Its Chemical and Mechanical Characteristics. Polymers.

[133] Raza Z.A., Abid S., Banat I.M. (2018). Polyhydroxyalkanoates: Characteristics, production, recent developments and applications. Int. Biodeterior. Biodegrad..

[134] Sabapathy P.C., Devaraj S., Meixner K., Anburajan P., Kathirvel P., Ravikumar Y., Zabed H.M., Qi X. (2020). Recent Developments in Polyhydroxyalkanoates (PHAs) Production—A Review. Bioresour. Technol..

[135] Tyagi P., Salem K.S., Hubbe M.A., Pal L. (2021). Advances in Barrier Coatings and Film Technologies for Achieving Sustainable Packaging of Food Products—A Review. Trends Food Sci. Technol..

[136] Yeo J.C.C., Muiruri J.K., Fei X., Wang T., Zhang X., Xiao Y., Thitsartarn W., Tanoto H., He C., Li Z. (2024). Innovative Biomaterials for Food Packaging: Unlocking the Potential of Polyhydroxyalkanoate (PHA) Biopolymers. Biomater. Adv..

[137] Turco R., Santagata G., Corrado I., Pezzella C., Di Serio M. (2020). In vivo and Post-synthesis Strategies to Enhance the Properties of PHB-Based Materials: A Review. Front. Bioeng. Biotechnol..

[138] Alfano S., Doineau E., Perdrier C., Preziosi-Belloy L., Gontard N., Martinelli A., Grousseau E., Angellier-Coussy H. (2024). Influence of the 3-Hydroxyvalerate Content on the Processability, Nucleating and Blending Ability of Poly(3-Hydroxybutyrate-co-3-hydroxyvalerate)-Based Materials. ACS Omega.

[139] Yu L.P., Yan X., Zhang X., Chen X.B., Wu Q., Jiang X.R., Chen G.Q. (2020). Biosynthesis of Functional Polyhydroxyalkanoates by Engineered Halomonas bluephagenesis. Metab. Eng..

[140] Singh S., Sithole B., Lekha P., Permaul K., Govinden R. (2021). Optimization of Cultivation Medium and Cyclic Fed-batch Fermentation Strategy for Enhanced Polyhydroxyalkanoate Production by Bacillus Thuringiensis Using a Glucose-rich Hydrolyzate. Bioresour. Bioprocess..

[141] Borrero-de Acuna J.M., Rohde M., Saldias C., Poblete-Castro I. (2021). Fed-Batch mcl- Polyhydroxyalkanoates Production in Pseudomonas putida KT2440 and DeltaphaZ Mutant on Biodiesel-Derived Crude Glycerol. Front. Bioeng. Biotechnol..

[142] Drieskens M., Peeters R., Mullens J., Franco D., Lemstra P.J., Hristova-Bogaerds D.G. (2009). Structure Versus Properties Relationship of Poly(lactic acid). I. Effect of Crystallinity on Barrier Properties. J. Polym. Sci. Pt. B-Polym. Phys..

[143] Auras R.A., Harte B., Selke S., Hernandez R. (2003). Mechanical, Physical, and Barrier Properties of Poly(Lactide) Films. J. Plast. Film Sheeting.

[144] Yamane K., Sato H., Ichikawa Y., Sunagawa K., Shigaki Y. (2014). Development of an Industrial Production Technology for High-molecular-weight Polyglycolic acid. Polym. J..

[145] Cabedo L., Luis Feijoo J., Pilar Villanueva M., Lagarón J.M., Giménez E. (2006). Optimization of Biodegradable Nanocomposites Based on aPLA/PCL Blends for Food Packaging Applications. Macromol. Symp..

[146] Messersmith P.B., Giannelis E.P. (1995). Synthesis and Barrier Properties of Poly(ε-caprolactone)-layered Silicate Nanocomposites. J. Polym. Sci. Pol. Chem..

[147] Yahiaoui F., Benhacine F., Ferfera-Harrar H., Habi A., Hadj-Hamou A.S., Grohens Y. (2015). Development of Antimicrobial PCL/Nanoclay Nanocomposite Films with Enhanced Mechanical and Water Vapor Barrier Properties for Packaging Applications. Polym. Bull..

[148] Xie L., Xu H., Chen J.-B., Zhang Z.-J., Hsiao B.S., Zhong G.-J., Chen J., Li Z.-M. (2015). From Nanofibrillar to Nanolaminar Poly(butylene succinate): Paving the Way to Robust Barrier and Mechanical Properties for Full-Biodegradable Poly(lactic acid) Films. ACS Appl..

[149] Sinha Ray S., Okamoto K., Okamoto M. (2003). Structure−Property Relationship in Biodegradable Poly(butylene succinate)/Layered Silicate Nanocomposites. Macromolecules.

[150] Charlon S., Follain N., Chappey C., Dargent E., Soulestin J., Sclavons M., Marais S. (2015). Improvement of Barrier Properties of Bio-based Polyester Nanocomposite Membranes by Water-assisted extrusion. J. Membr. Sci..

[151] Ren P.-G., Liu X.-H., Ren F., Zhong G.-J., Ji X., Xu L. (2017). Biodegradable Graphene Oxide Nanosheets/Poly-(butylene adipate-co-terephthalate) Nanocomposite Film with Enhanced Gas and Water Vapor Barrier Properties. Polym. Test.

[152] Jost V., Langowski H.-C. (2015). Effect of Different Plasticisers on the Mechanical and Barrier Properties of Extruded Cast PHBV Films. Eur. Polym. J..

[153] Crétois R., Follain N., Dargent E., Soulestin J., Bourbigot S., Marais S., Lebrun L. (2014). Microstructure and Barrier Properties of PHBV/Organoclays Bionanocomposites. J. Membr. Sci..

[154] Fabra M.J., Lopez-Rubio A., Lagaron J.M. (2014). Nanostructured Interlayers of Zein to Improve the Barrier Properties of High Barrier Polyhydroxyalkanoates and Other Polyesters. J. Food Eng..

[155] Siracusa V., Rocculi P., Romani S., Rosa M.D. (2008). Biodegradable Polymers for Food Packaging: A Review. Trends Food Sci. Technol..

[156] Satti S.M., Shah A.A. (2020). Polyester-based Biodegradable Plastics: An Approach Towards Sustainable Development. Lett. Appl. Microbiol..

[157] Thakur S., Chaudhary J., Sharma B., Verma A., Tamulevicius S., Thakur V.K. (2018). Sustainability of Bioplastics: Opportunities and Challenges. Curr. Opin. Green Sustain. Chem..

[158] Roohi, Kulsoom B., Mohammed K., Mohammed R.Z., Qamar Z., Mohammed F.K., Ghulam Md A., Anamika G., Gjumrakch A. (2017). Microbial Enzymatic Degradation of Biodegradable Plastics. Curr. Pharm. Biotechnol..

[159] Suresh V., Shams R., Dash K.K., Shaikh A.M., Béla K. (2025). Comprehensive Review on Enzymatic Polymer Degradation: A Sustainable Solution for Plastics. J. Agric. Food Res..

[160] Shin N., Kim S.H., Cho J.Y., Hwang J.H., Kim H.J., Oh S.J., Park S.-H., Park K., Bhatia S.K., Yang Y.-H. (2023). Fast Degradation of Polycaprolactone/Poly(butylene adipate-co-terephthalate) Blends by Novel Bacillus Strain NR4 with Broad Degrading Activity. J. Polym. Environ..

[161] Massardier-Nageotte V., Pestre C., Cruard-Pradet T., Bayard R. (2006). Aerobic and Anaerobic Biodegradability of Polymer Films and Physico-chemical Characterization. Polym. Degrad. Stabil..

[162] Atalay S.E., Bezci B., Özdemir B., Göksu Y.A., Ghanbari A., Jalali A., Nofar M. (2021). Thermal and Environmentally Induced Degradation Behaviors of Amorphous and Semicrystalline PLAs Through Rheological Analysis. J. Polym. Environ..

[163] Intergovernmental Panel on Climate Change (2023). Climate Change 2022—Impacts, Adaptation and Vulnerability: Working Group II Contribution to the Sixth Assessment Report of the Intergovernmental Panel on Climate Change.

[164] (2018). Standard Test Method for Determining Anaerobic Biodegradation of Plastic Materials Under High-Solids Anaerobic-Digestion Conditions.

[165] (2014). Plastics—Determination of the Ultimate Anaerobic Biodegradation Under High-Solids Anaerobic-Digestion Conditions—Method by Analysis of Released Biogas.

[166] (2018). Standard Test Method for Determining Aerobic Biodegradation of Plastic Materials in Soil.

[167] (2019). Plastics—Determination of the Ultimate Aerobic Biodegradability of Plastic Materials in Soil by Measuring the Oxygen Demand in a Respirometer or the Amount of Carbon Dioxide Evolved.

[168] (2021). Plastics—Soil Biodegradable Materials for Mulch Films for Use in Agriculture and Horticulture—Requirements and Test Methods Regarding Biodegradation, Ecotoxicity and Control of Constituents.

[169] (2021). Standard Test Method for Determining Aerobic Biodegradation of Plastic Materials Under Controlled Composting Conditions.

[170] (2012). Determination of the Ultimate Aerobic Biodegradability of Plastic Materials Under Controlled Composting Conditions—Method by Analysis of Evolved Carbon Dioxide—Part 1: General Method.

[171] (2023). Standard Specification for Labeling of Plastics Designed to be Aerobically Composted in Municipal and Industrial Aerobic Composting Facilities.

[172] (2021). Plastics—Organic Recycling—Specifications for Compostable Plastics.

[173] (2019). Plastics—Determination of the Ultimate Aerobic Biodegradability of Plastic Materials in an Aqueous Medium—Method by Measuring the Oxygen Demand in a Closed Respirometer.

[174] (2024). Standard Test Method for Determining Aerobic Biodegradation of Plastic Materials in the Marine Environment by a Defined Microbial Consortium or Natural Sea Water Inoculum.

[175] (2020). Plastics—Determination of Aerobic Biodegradation of Non-Floating Plastic Materials in a Seawater/Sediment Interface—Method by Analysis of Evolved Carbon Dioxide.

[176] (2016). Plastics—Determination of Aerobic Biodegradation of Non-Floating Plastic Materials in a Seawater/Sandy Sediment Interface—Method by Measuring the Oxygen Demand in a Closed Respirometer.

[177] (2021). Plastics—Determination of the Degree of Disintegration of Plastic Materials Under Defined Composting Conditions in a Pilot-Scale Test.

[178] Seo H., Hong H., Park J., Lee S.H., Ki D., Ryu A., Sagong H.Y., Kim K.J. (2025). Landscape Profiling of PET Depolymerases Using a Natural Sequence Cluster Framework. Science.

[179] Sun S. (2025). Enzyme-Embedded Biodegradable Plastic for Sustainable Applications: Advances, Challenges, and Perspectives. ACS Appl. Bio Mater..

[180] Guicherd M., Ben Khaled M., Guéroult M., Nomme J., Dalibey M., Grimaud F., Alvarez P., Kamionka E., Gavalda S., Noël M. (2024). An Engineered Enzyme Embedded into PLA to Make Self-biodegradable Plastic. Nature.

